# Shared and distinct gain-of-function consequences from pathologic cytoplasmic versus nuclear TDP-43

**DOI:** 10.1038/s41419-026-09143-9

**Published:** 2026-08-11

**Authors:** Aaron Long, Heather N. Currey, Matvey Goldberg, Randall J. Eck, Marina Han, Rebecca L. Kow, Brian C. Kraemer, Nicole F. Liachko

**Affiliations:** 1 Molecular and Cellular Biology Graduate Program, University of Washington, Seattle, WA USA; 2 Geriatrics Research Education and Clinical Center, Veterans Affairs Puget Sound Health Care System, Seattle, WA USA; 3 Graduate Program in Neuroscience, University of Washington, Seattle, WA USA; 4 Division of Gerontology and Geriatric Medicine, Department of Medicine, University of Washington, Seattle, WA USA; 5 Department of Psychiatry and Behavioral Sciences, University of Washington, Seattle, WA USA; 6 Department of Genome Sciences, Department of Medicine, University of Washington, Seattle, WA USA; 7 Department of Laboratory Medicine and Pathology, University of Washington, Seattle, WA USA

**Keywords:** Amyotrophic lateral sclerosis, Cell death in the nervous system

## Abstract

While predominantly nuclear localized in healthy cells, TDP-43 can transit between the nucleus and cytoplasm. In frontotemporal lobar degeneration (FTLD-TDP) and amyotrophic lateral sclerosis (ALS), TDP-43 accumulates in hyperphosphorylated cytoplasmic aggregates. However, a subset of patients develops nuclear aggregates. Expression of wild-type TDP-43 in *C. elegans* neurons results in nuclear protein accumulation and toxic gain of function. To enable comparative study of nuclear and cytoplasmic TDP-43 phenotypes, we generated new models with inactivating mutations in the nuclear localization sequence (NLS) of TDP-43. When compared to nuclear TDP-43 strains, similar protein levels of cytoplasmic TDP-43 cause less neuronal dysfunction in *C. elegans*. Using RNA sequencing, we determine that transcriptomes are largely unchanged in these models. However, we identify innate immune pathway increases in response to cytoplasmic but not nuclear TDP-43. We find phosphorylation-mediated neurotoxicity of both cytoplasmic and nuclear localized TDP-43 is controlled by the phosphatase calcineurin. We also find that overexpression of the unfolded protein response (UPR) transcription factor XBP-1s worsens outcomes for wild-type TDP-43 animals but improves TDP-43 ΔNLS, suggesting different pathways controlling toxicity and clearance of nuclear versus cytoplasmic TDP-43. Taken together, these data support shared and distinct cellular responses to nuclear versus cytoplasmic TDP-43 localization in an intact animal nervous system. This comparative approach supports a better understanding of human disease and informs therapeutic development for pathological subtypes of TDP-43 proteinopathies.

## Introduction

In 2006, TAR DNA-binding protein of 43 kDa (TDP-43) was identified as the major pathologic protein inclusion in the fatal neurodegenerative diseases amyotrophic lateral sclerosis (ALS) and frontotemporal lobar degeneration (FTLD-TDP) [1, 2]. ALS is characterized by loss of upper and lower motor neurons, which leads to progressive weakness of skeletal muscles, autonomic dysfunction, difficulty swallowing, speaking, and decreased respiratory function [3], while FTLD presents with degeneration in the frontal and anterior temporal lobes of the brain, resulting in social, emotional, and behavioral changes [4]. Following the discovery of TDP-43 inclusions in ALS and FTLD-TDP, TDP-43-positive inclusions were also found as a secondary pathology in Alzheimer’s disease (AD), the main cause of dementia globally [5]. The accumulation of TDP-43 aggregates in older adults, now termed limbic-predominant age-related TDP-43 encephalopathy (LATE), can be a frequent co-pathology in other neurodegenerative diseases, including Lewy body disease (LBD) and Huntington’s disease [6]. AD patients who have TDP-43 co-pathology have greater functional impairment, memory loss, poorer naming ability, and decreased hippocampal volume [7]. In many of these neurodegenerative diseases, TDP-43 is depleted from the nucleus and accumulates in the cytoplasm, where it undergoes post-translational modifications including hyperphosphorylation [8]. Some exceptions exist, with FTLD type A exhibiting both cytoplasmic and nuclear TDP-43 aggregates and FTLD type D having nuclear but not cytoplasmic TDP-43 aggregates [9]. Cytoplasmic mislocalization of TDP-43 results in a loss of its normal nuclear functions, while gaining new functions by interacting with or disrupting cytoplasmic pathways. Similarly, a recent study implicates nuclear TDP-43 pathology as an early event preceding cytoplasmic mislocalization [10]. How or why nuclear versus cytoplasmic TDP-43 aggregates occur, and whether they engage in similar pathways for disease pathogenesis and progression, remains to be determined.

In healthy cells, TDP-43 is ubiquitously expressed, predominantly nuclear, and controls many aspects of RNA metabolism. In particular, it regulates mRNA splicing of thousands of transcripts [11]. TDP-43 also participates in RNA transport, transcription, and translation [12]. In support of these roles, TDP-43 has protein domains including an N-terminal domain (NTD), nuclear localization signal (NLS), two RNA recognition motifs (RRM1 and RRM2), and a glycine-rich C-terminal domain thought to regulate protein-protein interactions. TDP-43 can undergo post-translational modifications, including oxidation, acetylation, phosphorylation, zinc binding, SUMOylation, ubiquitination, cleavage, and PARylation. Most of these post-translational modifications are only detected on aggregated TDP-43 in neurodegenerative disorders [13].

TDP-43 is actively imported into the nucleus and passively exported into the cytoplasm through the nuclear pore complex. In the nucleus, TDP-43 binds RNA with a preference for GU-rich sequences. Disrupting TDP-43 interactions with RNA by mutating the RNA-binding domains of TDP-43 or preventing RNA transcription, splicing, binding to other RNA-binding proteins, or export results in increased passive diffusion of TDP-43 through nuclear pore channels and the accumulation of TDP-43 in the cytoplasm [14]. The nuclear localization signal (NLS) of TDP-43 promotes active import of TDP-43 into the nucleus by binding to nuclear import adaptor Importin α1, which recruits the receptor Importin β to form an Importin α1/β/TDP-43 complex. Mutation of the TDP-43 NLS site abrogates the ability of TDP-43 to bind to Importin α1 and eliminates active transport into the nucleus [15, 16]. *C. elegans* express three Importin α homologs (IMA-1, IMA-2, and IMA-3) [17], which likely function similarly to promote TDP-43 nuclear localization.

TDP-43 is evolutionarily conserved, with a homolog in *C. elegans* designated TDP-1. TDP-1 is structurally similar to human TDP-43 but lacks a glycine-rich C-terminus. While in vertebrates TDP-43 is an essential gene [18–21], deletion of *C. elegans tdp-1* is tolerated, and does not result in apparent neuronal dysfunction. However, overexpression of TDP-1 results in an uncoordinated motor phenotype in *C. elegans* [22]. To better understand the role of TDP-43 in disease, transgenic models expressing human TDP-43 throughout the *C. elegans* nervous system have been generated and characterized [22–25]. Human TDP-43 has been shown to function appropriately in *C. elegans*, with genetic mutations causing similar dysfunction in *C. elegans* as in humans [26]. Expression of human TDP-43 in *C. elegans* causes progressive motor dysfunction, neuronal loss, a shortened lifespan, and the accumulation of insoluble, post-translationally modified TDP-43 in nuclear puncta without apparent cytoplasmic accumulation [22, 24, 27]. These models represent TDP-43 gain-of-function neurotoxicity, as their phenotypes do not recapitulate loss of *tdp-1* [24].

To examine differences in outcomes between pathological accumulation of TDP-43 in the nucleus versus the cytoplasm, we have generated and characterized new transgenic *C. elegans* strains that express human TDP-43 with point mutations inactivating the nuclear localization sequence (TDP-43 ΔNLS). We generated three TDP-43 ΔNLS strains with varying levels of TDP-43 protein in the cytoplasm. We then compared these strains to well-characterized *C. elegans* strains that express TDP-43 in the nucleus. Using neurofunctional, genetic and transcriptomic characterizations, we find shared and distinct pathogenic outcomes between nuclear versus cytoplasmic localized TDP-43. These models allow exploration of TDP-43 gain-of-function effects in the absence of loss-of-function, enabling clear dissection of pathogenic consequences with relevance to human ALS and FTLD-TDP.

## Materials and methods

### Strains and transgenics

Wild-type (non-Tg) *C. elegans* (Bristol strain N2) was maintained as previously described [28]. A plasmid carrying the human TDP-43 coding sequence downstream of the pan-neuronal *C. elegans snb-1* promoter underwent site-directed mutagenesis (Agilent Quikchange) to incorporate three point mutations K82A/R83A/K84A [29], generating TDP-43 ΔNLS. As previously described [30], multicopy integrated transgenes were produced by microinjection of the transgene marker and the transgene into the germline of N2 adults, followed by integration and outcrossing. Three strains, NLS152, NLS151, and NLS142, were categorized by TDP-43 protein level into TDP-43 ΔNLS (low), TDP-43 ΔNLS (med), and TDP-43 ΔNLS (high).

Additional strains used include CK410 (TDP-43-Tg), CK403 (TDP-43-Tg 2), CK402 (TDP-43-Tg 3), CK1943 (TDP-43-Tg (low), CK423 (TDP-43 M337V), AGD927 (xbp-1s o/ex), RB1667 (tax-6-KO), KJ306 (tax-6 GOF), CK3053 (tdp-1 KO), VC3056 (zip-2(ok3730), and CZ1200 (Control [Unc-25p::GFP]). CK410, CK403, CK402, CK1943, and CK423 use a P_snb-1_ pan-neuronal promoter to drive human wild-type TDP-43 or TDP-43 M337V expression. The strain CZ1200 (a gift from Dr. Y. Jin, University of California, San Francisco, CA) has an integrated p_unc-25_::GFP transgene expressed in GABAergic neurons that marks cell bodies and axons [31]. NLS152, NLS151, and NLS142 were crossed with CZ1200 to generate TDP-43 ΔNLS low, medium, and high strains that have GFP-labeled GABAergic neurons. CK144 uses the P_aex-3_ pan-neuronal promoter to drive expression of human WT Tau (4R1N). AGD927 expresses constitutively active XPB-1s under a P_rab-3_ pan-neuronal promoter. AGD927 was crossed with nuclear and cytoplasmic TDP-43 strains as well as a tau strain. See Table S1 for a complete list of all strains used in this study.

### Immunoblotting

Stage-matched day 1 adult *C. elegans* were harvested into 50μl pellets and flash frozen in liquid nitrogen. Pellets were resuspended in 1x sample buffer, sonicated 3 × 60% amplitude for 10 s, and boiled for 10 min. 4-10μl of samples were loaded into 4–15% Criterion™ Tris-HCl Protein Gels (BioRad), and transferred onto PVDF membrane. TDP-43 was detected by monoclonal Anti-TDP43 antibody (ab190963[EPR18554], Abcam, 1:1000), pTDP-43 was detected by monoclonal Anti-phospho TDP-43 (pS409/410, CAC-TIP-PTD-M01A, CosmoBio, 1:1000), tubulin was detected by monoclonal antibody E7 (Developmental Studies Hybridoma Bank, 1:5000). Secondary antibodies used were Sheep anti-Mouse IgG peroxidase-linked whole antibody Secondary Antibody (Cytiva, 45-000-692, 1:5000) and Donkey anti-Rabbit IgG, peroxidase-linked species-specific whole antibody Secondary Antibody (Cytiva, 45-000-682, 1:10000). Signal was visualized on film using ECL (Clarity Western ECL Substrate 1705061) in a 1:1 ratio for up to 15 min. Quantification was completed in ImageJ using densitometry of scanned film. Full immunoblots for all figures are included as Supplemental Material.

### C. elegans immunohistochemistry

Slides were coated with poly-L-lysine and baked at 60 °C for 40 min. Day 1, adult animals were washed with M9 and then transferred into a 5 μl droplet of 1% PFA. After 5 min, animals were compressed under a cover slip. Slides were left on a dry-ice chilled metal block for 30 min. The cover slip was quickly removed to tear away upper levels of *C. elegans* cuticle, and slides were dipped in ice-cold methanol for 5 min, followed by acetone for 5 min. Slides were entered into a humidified chamber for block, antibody incubation steps, and counterstain. Fixed whole animals were stained with anti-TAR DNA-binding protein 43 (TDP-43) (AbCam, ab57105) at a titer of 1:500. ALEXA 647-conjugated anti-mouse antibody was used as the secondary antibody at a titer of 1:1000. Slides were counterstained with 300 nM 4,6-diamidino-2-phenylindole (DAPI) nuclear stain in PBS. Images were acquired on an Andor Dragonfly 200,100 × oil immersion lens (Andor UK, Belfast). Images were processed in FIJI: ImageJ from 30.72 × 30.72 micron (512 × 512) ROI OEM TIFF files.

### Motility assays

Radial locomotion (crawling) and thrashing (swimming) assays were conducted on unstimulated staged-matched day 1 adult *C. elegans* at room temperature as previously described [32]. In brief, for an individual radial locomotion assay, 10–15 worms were placed in the center of a 10 cm NGM plate with OP-50 bacteria (food source) and allowed to crawl freely on the plate for an hour, and the linear distance was measured from the starting point. Radial locomotion assays were conducted in duplicate on an individual testing day (*n* = 20–30), and with three independent testing days (*N* = 3), for a total *n* = 60–90 animals. For thrashing assays, roughly 50 *C. elegans* were transferred in 1 ml M9 buffer onto 35 mm NGM assay plates. Worms were recorded for 1 min at 14 frames per second by the WormLab system (MBF Bioscience). Swimming tracks were verified post-recording and repaired as needed. Thrashing assays were conducted on three independent testing days (*N* = 3), for an estimated total *n* = 150 animals. Figures from radial locomotion and thrashing are from at least three independent biological replicates per genotype.

### Neurodegeneration

Live day 1 adult worms were placed on a 3% agarose pad and immobilized with 0.01% sodium azide. GABAergic neurons were counted using fluorescence microscopy using a 60x objective on an upright microscope (Leica DM4 B). Scoring was conducted blinded to the genotype. Neuronal death, axonal defasciculation, gaps in the continuity of the nerve cord, and non-neuronal puncta were scored as signs of neurodegeneration. Figures are from at least three independent replicates. Representative images of axonal defasciculation, gaps in the continuity of the nerve cord, were taken using the Andor Dragonfly at 100x and processed in FIJI.

### RNA extraction

RNA was extracted from snap-frozen *C. elegans* pellets using the Thermo Fisher Scientific TRIzol RNA extraction protocol. RNA was resuspended in nuclease-free water, run on an agarose gel to assess integrity, and concentration was assessed using a NanoPhotometer NP80 spectrophotometer (Implen GMbH).

### qRT-PCR

Day 1 adult *C. elegans* cDNA was synthesized from RNA using iScript Reverse Transcription Supermix for RT-qPCR (Bio-Rad) and a T100 Thermal Cycler (Bio-Rad). Controls include no-RT supermix without the reverse transcriptase enzyme, and no template reaction mix. cDNA samples and no-RT controls were mixed with SYBR supermix and primers for TDP-43, *act-1*, or *rpl-32* and run on a CFX Connect RT-PCR Detection System (Bio-Rad). The strain CK423 was used to make a standard curve, using concentrations of 100 ng, 10 ng, 1 ng, and 0.1 ng of cDNA. All experimental samples and controls were run at 10 ng of cDNA per well.

### RNA sequencing

RNA extracted from the L4 stage *C. elegans* was assembled into poly-A-enriched libraries and sequenced at a read-depth of 25 million pair-end reads per sample as a fee-for-service (Novogene). FASTQ files were pre-processed with FASTP (version 0.23.4) [33], aligned with STAR two-pass-mapping (version 2.7.10b) [34], and quantified with featureCounts (version 2.20.0) in R (version 4.4.2) without multi-mapping reads [35] against the *C. elegans* genome WBcel235 (version 113) [36]. Differential gene expression was calculated with DESeq2 (version 1.46.0) [37] in R (version 4.4.2) [38]. Transcription factor activity was estimated with CelEsT (version 1.0) [39] in R (version 4.4.2) [38] using decoupleR (version 2.12.0) [40] and the recommended database of TF-target pairs from previously generated differential gene expression data. Differential mRNA splicing was calculated for both annotated and novel splicing sites with SpliceWiz (version 1.8.0)[41] and DESeq2 (version 1.46.0) [37] in R (version 4.4.2) [38] with default SpliceWiz filters. All visualizations were created with ggplot2 (version 3.5.1) [42] in R (version 4.4.2) [38].

### Statistical analysis

All data were analysed using GraphPad Prism software, unless otherwise described. Statistical significance was determined using one-way ANOVA with Tukey’s multiple comparison test. Error bars on graphs represent the standard error of the mean. *P*-values were expressed as: * (*p* < 0.05), **(*p* < 0.01), ***(*p* < 0.001), ****(*p* < 0.0001) and ns is not statistically significant (*p* > 0.05).

## Results

**Fig. 1:**
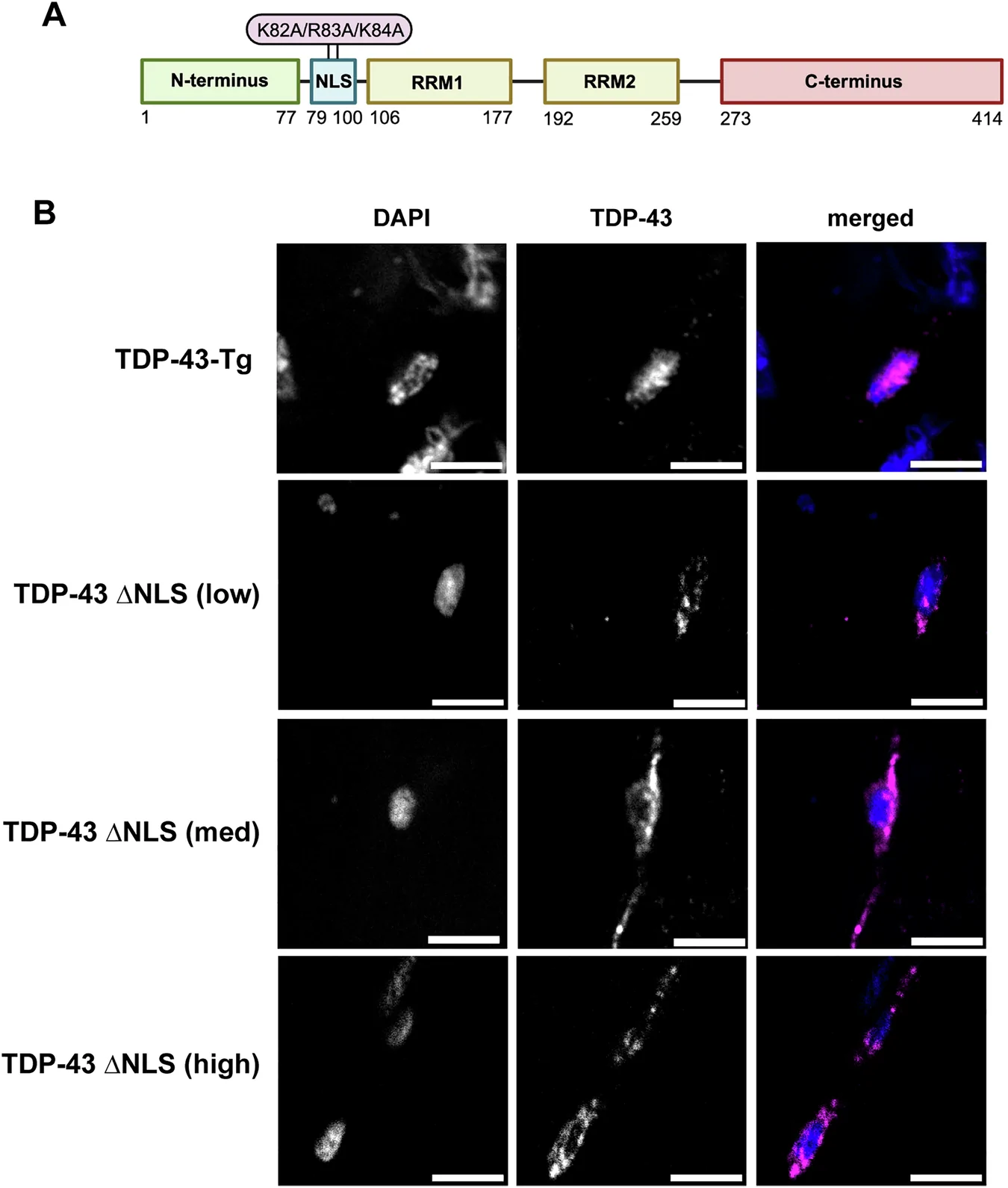
TDP-43 is localized in the cytoplasm and not the nucleus in TDP-43 ΔNLS worms. **A** TDP-43 protein domains, including the N-terminus, nuclear localization signal (NLS), RNA recognition motifs (RRM1 and RRM2), and C-terminus. The location of inactivating point mutations within the NLS is shown. **B** Immunostaining for nuclei (DAPI) and total TDP-43 in the day 1 adult nerve cord of TDP-43-Tg, and TDP-43 ΔNLS (low), TDP-43 ΔNLS (medium), and TDP-43 ΔNLS (high) *C. elegans*. Size bar = 10μm.

It has been previously shown that wild-type human TDP-43 expressed in *C. elegans* (TDP-43-Tg) is strongly localized to the nucleus [24]. To characterize differences between the gain-of-function consequences of nuclear versus cytoplasmic TDP-43, we developed new *C. elegans* models with cytoplasmic-localized TDP-43, referred to as TDP-43 ΔNLS. TDP-43 ΔNLS was created by adding three point mutations (K82A/R83A/K84A) in the nuclear localization sequence of TDP-43, consistent with mammalian TDP-43 ΔNLS models generated previously [16, 43] (Fig. 1A). Using this strategy, we established three TDP-43 ΔNLS strains with differing levels of transgene expression referred to as TDP-43 ΔNLS (low), TDP-43 ΔNLS (med), and TDP-43 ΔNLS (high).

### TDP-43 ΔNLS is localized to the cytoplasm

To confirm localization of TDP-43 in the TDP-43 ΔNLS strains, we performed TDP-43 immunohistochemistry with nuclear counterstaining in four strains: TDP-43-Tg (expresses wild-type human TDP-43), TDP-43 ΔNLS (low), TDP-43 ΔNLS (med), and TDP-43 ΔNLS (high) (Fig. 1B). In these strains, TDP-43 ΔNLS (low), TDP-43 ΔNLS (med), and TDP-43 ΔNLS (high) appeared predominantly cytoplasmic, more closely resembling the cytoplasmic distribution of TDP-43 in human disease, while TDP-43-Tg was strongly nuclear, consistent with previously published results (Fig. 1B and Supplementary Fig. 1).

### TDP-43 ΔNLS strains exhibit dose-responsive neurotoxicity

**Fig. 2:**
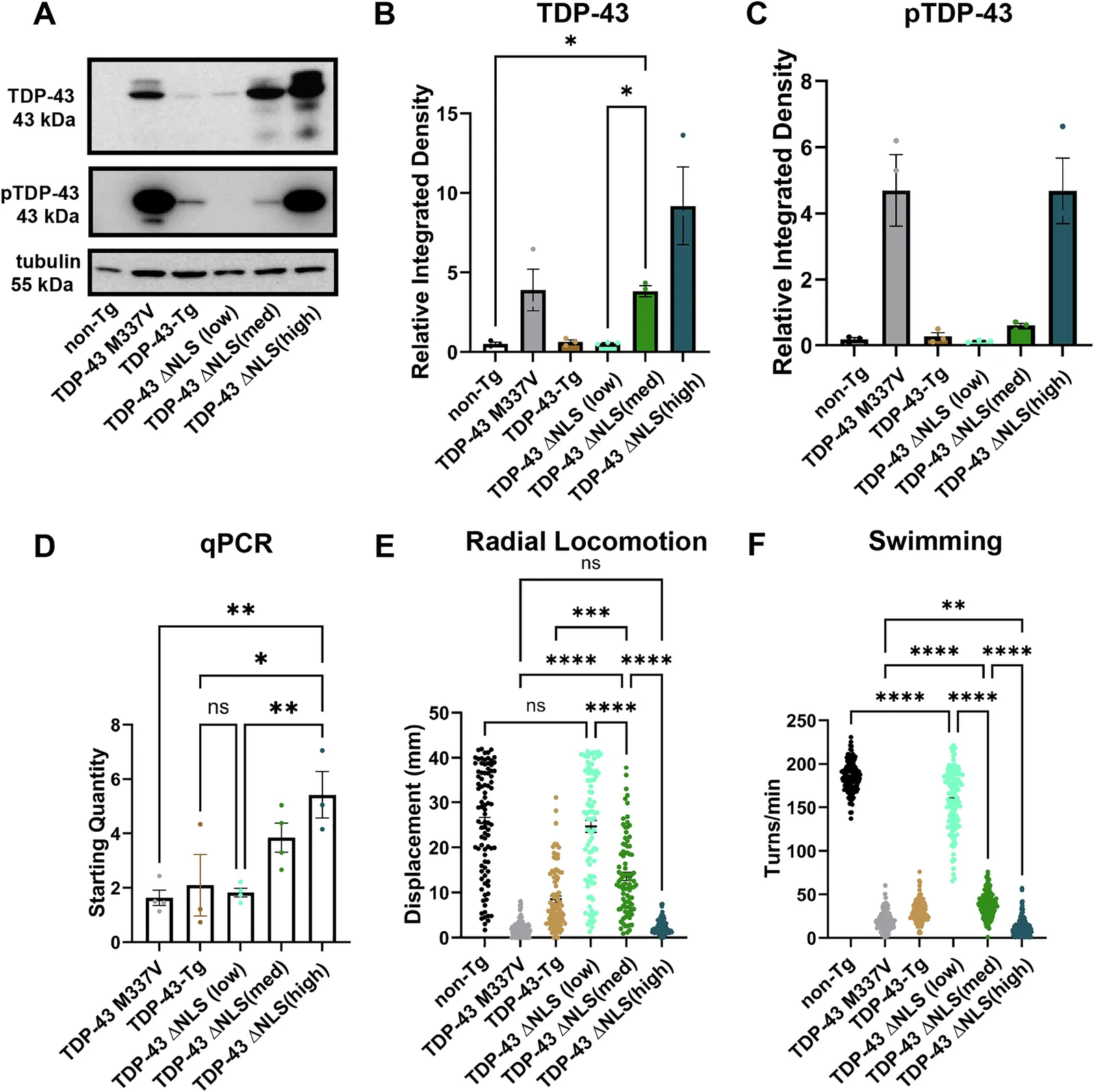
TDP-43 ΔNLS causes dose-dependent motility impairment. **A** Synchronized day 1 adult *C. elegans* were tested by immunoblot for total TDP-43, phosphorylated TDP-43, and tubulin (load control). The immunoblot is representative of three independent biological replicates. **B**, **C** Quantification of protein levels from scanned film images. *p < 0.05 **D** qRT-PCR of TDP-43 expression in day 1 adult *C. elegans*. *act-1* mRNA was used as an internal expression control. TDP-43 mRNA was highest in the TDP-43 ΔNLS (high) strain. *p < 0.05, **p < 0.01. Motility impairment of TDP**-**43 ΔNLS as measured by (**E**) radial locomotion (linear distance crawling in one hour on an agar plate) or **F** swimming (rates of thrashing in liquid for a minute). Higher levels of cytoplasmic TDP-43 resulted in decreased crawling and fewer turns/minute. One-way ANOVA with Tukey’s multiple comparison test. **p < 0.01, ***p < 0.001, ****p < 0.0001. ns, not significant. Data are mean ± s.e.m.

We performed several measures to characterize TDP-43 ΔNLS strains. To determine expression levels of TDP-43, we performed immunoblot analyses on stage-matched day 1 adult populations of animals. We determined the three TDP-43 ΔNLS strains had varying levels of TDP-43 expression and were designated TDP-43 ΔNLS (low), TDP-43 ΔNLS (med), and TDP-43 ΔNLS (high) based on detected protein (Fig. 2A–C and Supplementary Fig. 2). As comparators, we included well-characterized *C. elegans* strains expressing wild-type TDP-43 (TDP-43-Tg) or familial ALS (fALS) mutant TDP-43 (TDP-43 M337V) [24]. We found that TDP-43 ΔNLS (low) had low levels of TDP-43 that were clearly detected upon overexposure (Supplementary Fig. 2). There was no detectable pTDP-43 in TDP-43 ΔNLS (low) upon overexposure (data not shown). TDP-43 ΔNLS (med) had similar levels of TDP-43 as TDP-43 M337V but much less pTDP-43. TDP-43 ΔNLS (high) had roughly twice as much TDP-43 as TDP-43 M337V and comparable levels of pTDP-43 as TDP M337V (Fig. 2A–C).

We also determined mRNA expression levels of TDP-43 ΔNLS (low), TDP-43 ΔNLS (med), and TDP-43 ΔNLS (high) using quantitative reverse transcription PCR (qRT-PCR) (Fig. 2D). mRNA levels of the TDP-43 ΔNLS strains followed similar trends as protein quantity, with increasing levels of TDP-43 mRNA corresponding to higher amounts of protein detected (Fig. 2A–D).

### Neuronal dysfunction corresponds to the expression level of TDP-43 ΔNLS

Motility of *C. elegans* can serve as a sensitive readout of neuronal function [44] and has been used to quantify neuronal dysfunction and neurodegeneration in models of neurodegenerative diseases [45, 46]. To quantify motility defects in TDP-43 ΔNLS strains, we performed two assays: radial locomotion for assessment of the ability to crawl on a solid agar surface, and swimming testing the ability to thrash in liquid [32, 47]. Radial locomotion and swimming both give a readout of GABAergic and cholinergic neurons, as well as associated circuits responsible for movement initiation, propagation, and cessation [32, 46].

For both radial locomotion and swimming, our TDP-43 ΔNLS strains had an inverse relationship with TDP-43 protein levels; with TDP-43 ΔNLS (low) having near normal motility, TDP-43 ΔNLS (med) having moderate impairment, and TDP-43 ΔNLS (high) having severe impairment (Fig. 2E, F). Surprisingly, TDP-43 ΔNLS (med) had similar crawling and swimming as TDP-43-Tg (Fig. 2E, F) even though the strain has significantly more total TDP-43 protein (Fig. 2A, B). Likewise, TDP-43 ΔNLS (high) had similar crawling and swimming as TDP-43 M337V (Fig. 2E, F) despite accumulating more TDP-43 protein (Fig. 2B, C). However, levels of pTDP-43 are comparable between TDP-43 ΔNLS (med) and TDP-43-Tg, and between TDP-43 ΔNLS (high) and TDP-43 M337V, which may support previous findings that abundance of pTDP-43 correlates with neurotoxicity in *C. elegans* [24].

To examine whether the endogenous *C. elegans* homolog of TDP-43, *tdp-1*, contributes to motility defects in TDP-43 ΔNLS, we crossed *tdp-1(bk3053)*, a CRISPR-generated whole-gene deletion of *tdp-1*, with a TDP-43 ΔNLS (med) and TDP-43-Tg 3. However, we did not see any significant differences in motility in the absence of *tdp-1* (Supplementary Fig. 3).

**Fig. 3:**
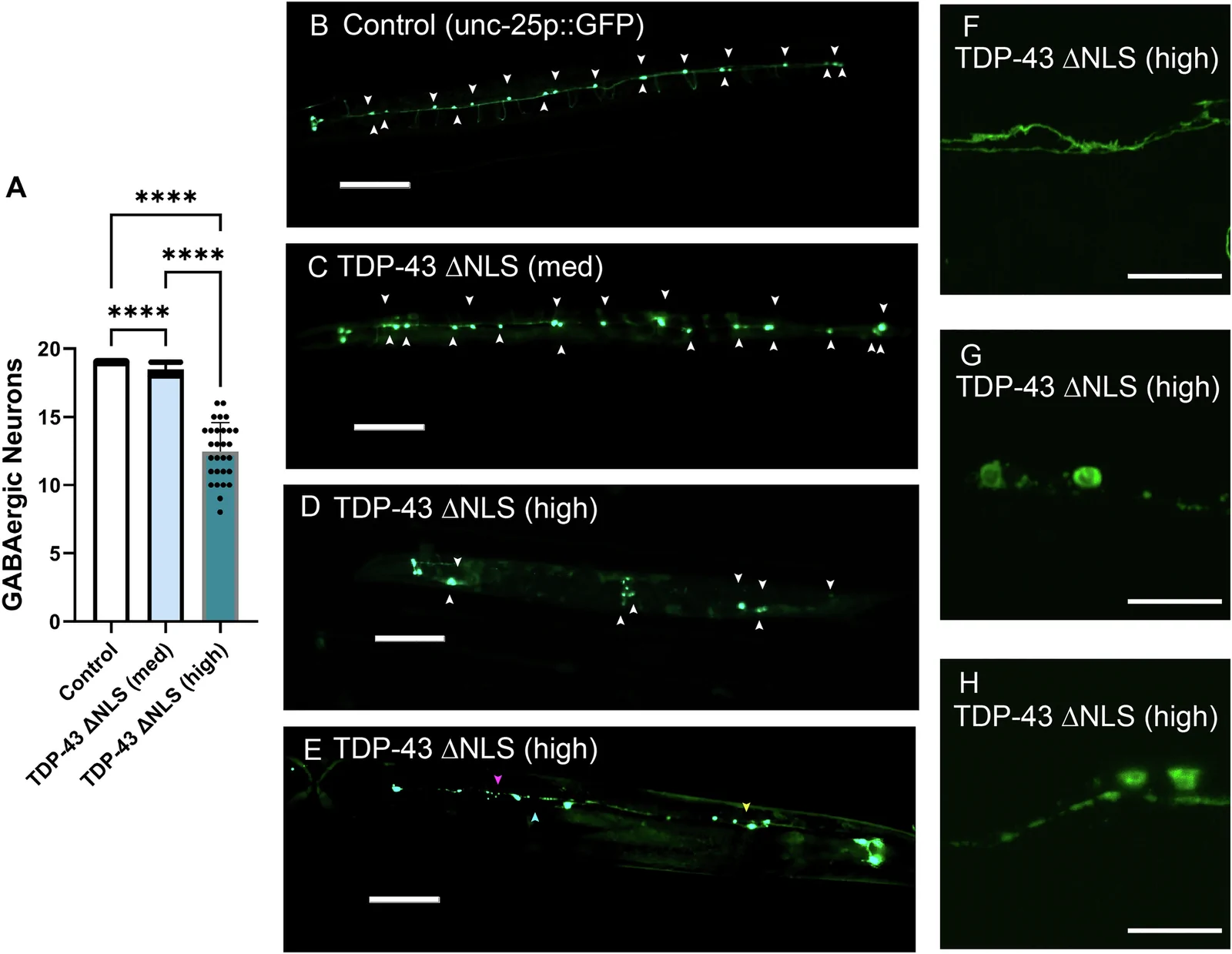
TDP-43 ΔNLS causes loss of GABAergic neurons in a dose dependent manor. GABAergic neurons were counted in vivo in TDP-43 ΔNLS (high) and TDP-43 ΔNLS (med) strains using an *unc-25p::GFP* reporter. Neuronal death, axonal defasciculation, gaps in the continuity of the nerve cord, and non-neuronal puncta along the nerve cord were scored signs of neurodegeneration. **A** Number of GABAergic neurons present, with TDP-43 ΔNLS (med) (*n* = 20) and TDP-43 ΔNLS (high) (*n* = 19) having respectively 0.5 and 6.5 neurons less than the control (*n* = 30). A representative nerve cord in control (**B**), TDP-43 ΔNLS (med) (**C**), and TDP-43 ΔNLS (high) (**D**). White arrows point to each GABAergic neuron. Scale bar = 80μM. **E** Examples of the neurodegenerative changes in a TDP-43 ΔNLS (high) worm. The yellow arrows point at axonal defasciculation, the magenta arrow points at non-neuronal puncta, and the turquoise arrow points at gaps in the nerve cord. Scale bar = 40μM. Representative images of axonal defasciculation (**F**), non-neuronal puncta (**G**), and gaps in the nerve cord (**H**) in TDP-43 ΔNLS (high). Scale bar = 10μM.

### High levels of cytoplasmic phosphorylated TDP-43 cause neurodegeneration

TDP-43 ΔNLS (med) and TDP-43 ΔNLS (high) have clear motility defects, indicating possible neurodegeneration. GABAergic neurons control *C. elegans* motility [48] and are vulnerable to TDP-43-Tg and TDP-43 M337V [24]. To examine the integrity of GABAergic neurons in TDP-43 ΔNLS, we used a transgenic reporter that expresses GFP in the 19 GABAergic neurons (*unc-25p::GFP*) to visualize and quantify changes in GABAergic neurons [24]. *C. elegans* have 19 D-type GABAergic motor neurons. Control animals (*unc-25p::GFP*) had intact GABAergic neurons and axons with a continuous nerve cord, no non-neuronal puncta, and no visual signs of neurodegeneration (Fig. 3A, B). TDP-43 ΔNLS (med) worms appeared similar to controls, but about half of the worms had one fewer GABAergic neuron (Fig. 3A, C). However, TDP-43 ΔNLS (high) animals exhibited much more neurodegeneration, with an average of 6.5 fewer GABAergic neurons than the control (Fig. 3A, D). In addition to loss of GABAergic neurons, we also saw axonal defasciculation, gaps in the continuity of the nerve cord, and non-neuronal puncta along the nerve cord in all TDP-43 ΔNLS (high) worms analyzed (Fig. 3E–H).

### Transcriptome differences between models of cytoplasmic versus nuclear TDP-43

**Fig. 4:**
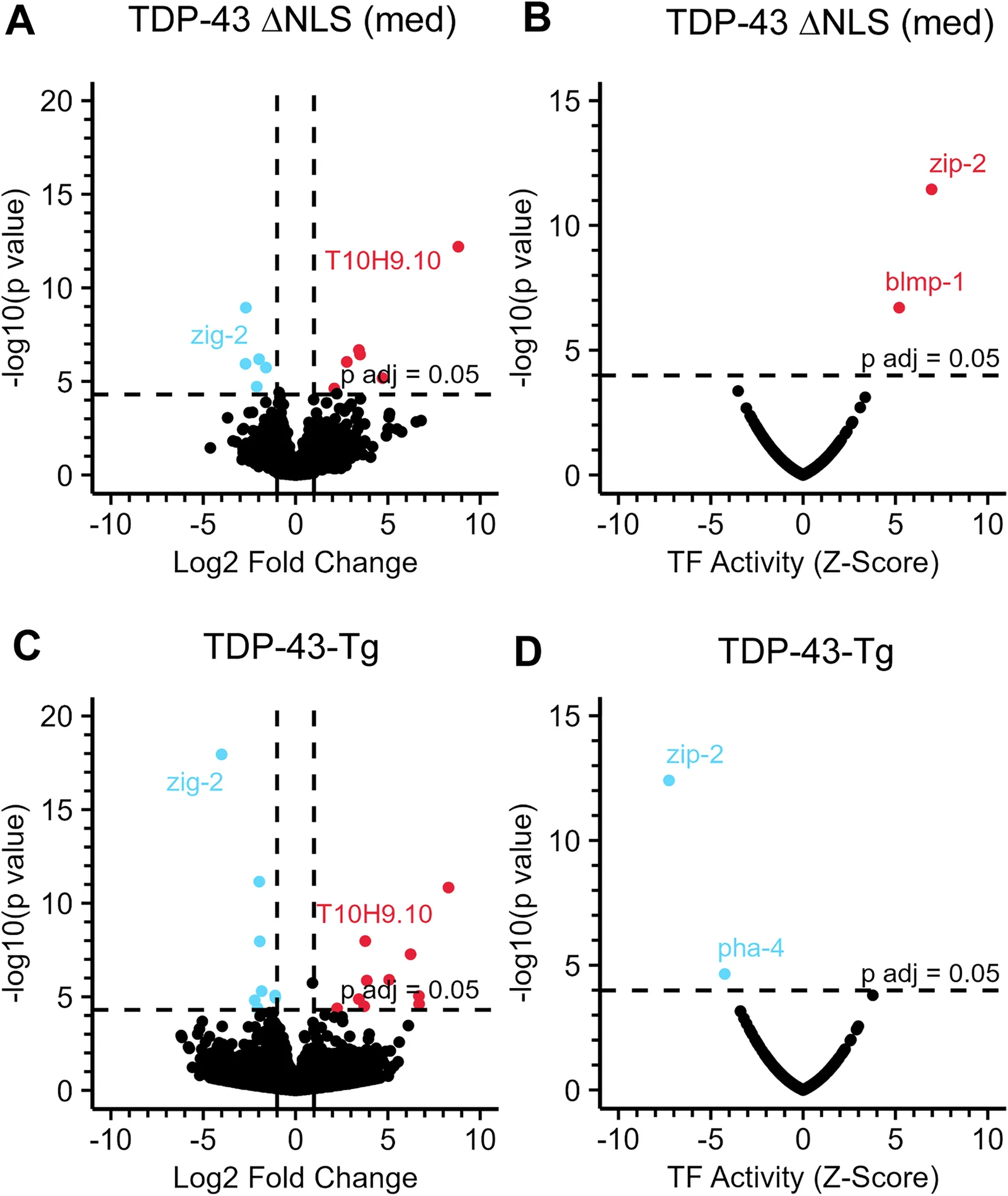
Differential gene expression in nuclear and cytoplasmic TDP-43 strains. **A** A volcano plot of differential gene expression in TDP-43 ΔNLS (med) Tg animals compared to wildtype controls (*n* = 8). **B** A volcano plot of estimated differential transcription factor activity in TDP-43 ΔNLS (med) Tg animals compared to non-Tg controls (*n* = 8). **C** A volcano plot of differential gene expression in TDP-43-Tg animals compared to non-Tg controls (*n* = 8). **D** A volcano plot of estimated differential transcription factor activity in TDP-43-Tg animals compared to non-Tg controls (*n* = 8). **A–D** Dotted line represents the threshold for p adj = 0.05.

To determine the molecular consequences of nuclear and cytoplasmic TDP-43 in *C. elegans*, we performed RNA sequencing on polyA-enriched RNAs from TDP-43-Tg and TDP-43 ΔNLS (med) at the L4 stage. Overall, there were relatively few transcriptomic differences among TDP-43 ΔNLS, TDP-43-Tg, or N2 controls. These results are consistent with principal component analysis (PCA) where the groups are largely overlapping (Supplementary Fig. 4A). However, compared to stage-matched non-Tg controls, TDP-43 ΔNLS (med) animals significantly differentially expressed 11 genes (p adj < 0.05 & |log2 fold change | > 1.0) (Fig. 4A and Table S2) including the downregulation of immunoglobulin *zig-2*, which can be secreted from neurons to influence axon maintenance and interact with insulin [49, 50]. To gain insight into the compensatory or effector cellular signals initiated by cytoplasmic TDP-43 aggregation and responsible for driving gene expression changes, we performed transcription factor (TF) network analysis to estimate significantly differentially active TFs using the computational tool CelEst (p adj < 0.05) [39]. We found an upregulation of predicted target genes for the stress and infection-sensing TF ZIP-2 [51] and cell-death regulator BLMP-1 [52] in TDP-43 ΔNLS (med) animals (Fig. 4B and Table S3).

We also performed RNA sequencing on TDP-43-Tg animals, which accumulate nuclear soluble and insoluble TDP-43. We found TDP-43-Tg animals significantly differentially expressed 19 genes (padj < 0.05) relative to non-Tg controls (Fig. 4C and Table S4), sharing five with TDP-43 ΔNLS (med) animals, including *zig-2*. We estimated 2 TFs had significantly decreased activity in TDP-43 animals (Fig. 4D and Table S5), including ZIP-2 and the forkhead box PHA-4. Notably, ZIP-2’s activity is reversed compared to that in TDP-43 ΔNLS (med) animals.

To examine the inverse relationship between estimated ZIP-2 transcription factor activity in TDP-43 ΔNLS versus TDP-43-Tg, we crossed a partial deletion of the *zip-2* coding sequence, *zip-2(ok3730),* into these strains. We tested whether ZIP-2 transcription factor activity modifies TDP-43 phenotypes by evaluating the motility of TDP-43 ΔNLS; zip-2(ok3730) and TDP-43-Tg; zip-2(ok3730). However, we found that there were no significant differences between these strains and controls (Supplementary Fig. 5), suggesting that ZIP-2 transcription factor activity is not promoting either neuroprotective or neurodegenerative outcomes.

**Fig. 5:**
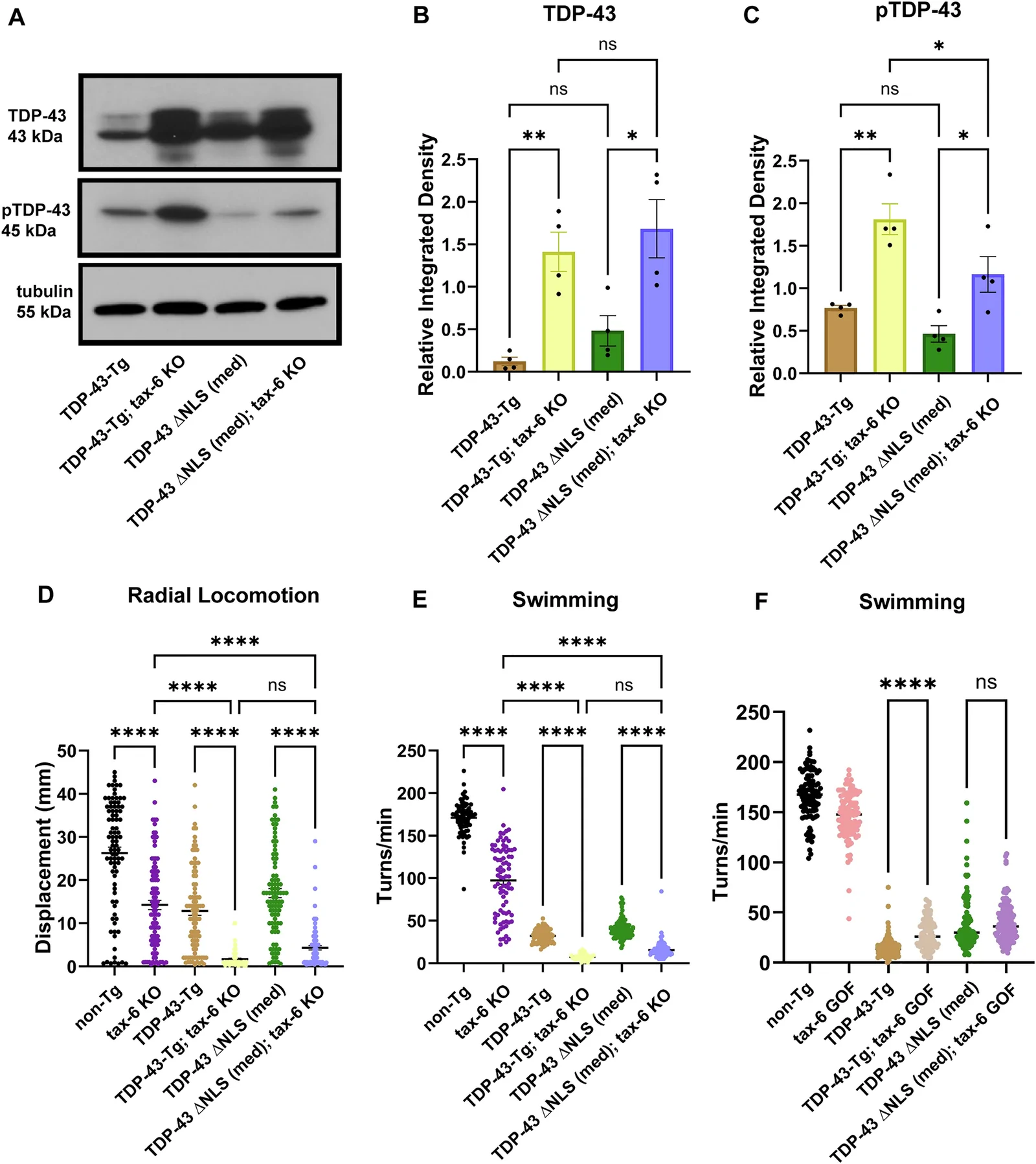
*tax-6* controls TDP-43 phosphorylation levels in both nuclear and cytoplasmic TDP-43 strains. **A** Synchronized populations of day 1 adult *C. elegans* were tested by immunoblot for total TDP-43, phosphorylated TDP-43, and tubulin (load control). Immunoblots shown are representative of four independent biological replicates. **B**, **C** Quantification of protein levels from four independent biological replicates. *p < 0.05, **p < 0.01. ns, not significant. *tax-6 KO* day 1 adult motility as measured by **D** radial locomotion (linear distance crawling in one hour on an agar plate) or **E** swimming (rates of thrashing in liquid for a minute). TDP-43-Tg and TDP-43 ΔNLS (med) strains were both equally affected by *tax-6 KO*. **F** *tax-6 GOF* motility measured by swimming. Motility impairment of TDP-43-Tg but not TDP-43 ΔNLS (med) strains was partly rescued by tax-6 GOF. One-way ANOVA with Tukey’s multiple comparison test. ****p < 0.0001. ns, not significant. Data are mean ± s.e.m.

To identify the relative contribution of gain-of-function and loss-of-function mechanisms to TDP-43 toxicity in *C. elegans*, we also performed RNA splicing analysis on our RNA sequencing from TDP-43 ΔNLS (med) and TDP-43-Tg animals, and on previously published RNA sequencing from *tdp-1(csb38)* null animals [53]. We found that TDP-43 ΔNLS (med) and TDP-43-Tg animals did not significantly alter the splicing of any events (p adj < 0.05 & |deltaPSI | > 0.1) (Supplementary Fig. 4B, C and Tables S6–7). Consistent with known effects of TDP-43 loss of function, the loss of *tdp-1* did alter splicing, including exon retention events (Supplementary Fig. 4D and Table S8). The excessive activity of nuclear TDP-43 is associated with an increase in exon-skipping [54], which we did not observe in TDP-43-Tg animals. Taken together, these transcriptomic analyses suggest both shared and distinct cellular responses to the presence of cytoplasmic versus nuclear TDP-43 aggregation. These data also suggest that the molecular mechanisms for TDP-43-driven neurodegeneration in these models are predominantly independent of RNA metabolism.

### Calcineurin phosphatase activity protects against both nuclear and cytoplasmic TDP-43

Phosphorylation of TDP-43 S409/S410 is consistently present in familial and sporadic TDP-43 proteinopathies [55], and in *C. elegans* potentiates worsened motility [24]. The phosphatase calcineurin directly binds and dephosphorylates TDP-43, reducing the accumulation of phosphorylated TDP-43 [56]. *C. elegans* has a single homolog of calcineurin, TAX-6. Loss-of-function mutations in *tax-6* result in severely worsened motility defects in TDP-43-Tg and TDP-43 M337V [56]. To test whether calcineurin/*tax-6* similarly influences cytoplasmic TDP-43, we crossed *tax-6(ok2065)* to TDP-43-Tg and TDP-43 ΔNLS (med). In the absence of functional *tax-6*, both TDP-43-Tg and TDP-43 ΔNLS (med) strains had significantly increased total and phosphorylated TDP-43, worsened radial locomotion and swimming (Fig. 5A–E). These data support TAX-6-mediated protection against phosphorylation-driven neurotoxicity of both cytoplasmic and nuclear TDP-43.

We also tested whether a constitutively active *tax-6* gain-of-function mutant, tax-6 GOF, could protect against TDP-43-Tg and TDP-43 ΔNLS (med) motility defects. We found that this was able to partly rescue TDP-43-Tg but not TDP-43 ΔNLS (med) (Fig. 5F), which may reflect differences in constitutive TAX-6 activity in the nucleus versus cytoplasm, differing contributions of phosphorylated TDP-43 to toxicity in TDP-43-Tg versus TDP-43 ΔNLS, or variability due to the relatively low levels of phosphorylated TDP-43 at baseline in these strains.

### XBP-1s differentially modifies cytoplasmic versus nuclear TDP-43 in *C. elegans* neurotoxicity

**Fig. 6:**
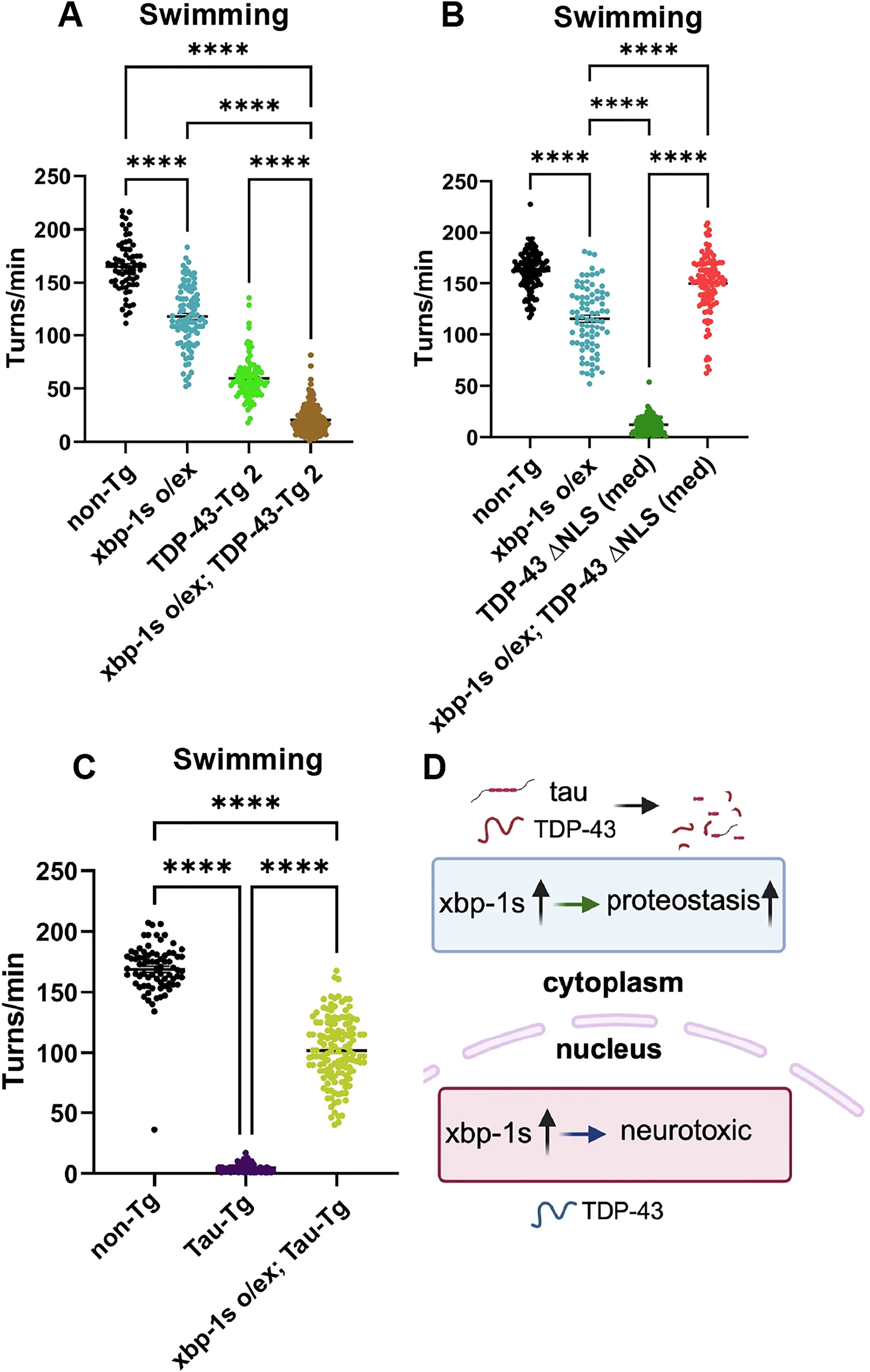
*xbp-1s* overexpression protects against cytoplasmic TDP-43 but worsens outcomes for nuclear TDP-43 strains. *xbp-1s o/e* driven motility changes of day 1 adults as measured by swimming (rates of thrashing in liquid for a minute). *xbp-1s o/ex* **A** worsened swimming in TDP-43-Tg-2 but rescued swimming in **B** TDP-43 ΔNLS (med) and **C** tau-Tg. **D** Schematic of XBP-1s activation outcomes targeting nuclear versus cytoplasmic TDP-43. Increasing XBP-1s increases proteostasis to protect against TDP-43 and tau in the cytoplasm, but leads to increased neurotoxicity when TDP-43 is nuclear. Higher levels of XBP-1s are only beneficial when dealing with cytoplasmic proteinopathies. One-way ANOVA with Tukey’s multiple comparison test. ****p < 0.0001. Data are mean ± s.e.m.

Proteostatic endoplasmic reticulum (ER) stress initiates the unfolded protein response (UPR^ER^), resulting in expression of the active form of the transcription factor X-box binding protein, XBP-1s [57]. In aging *C. elegans*, expression of XBP-1s activates the UPR^ER^, enables resistance to ER stress, and extends lifespan [58]. In some neurodegenerative disease models, XBP-1 activation triggers the UPR^ER^ protecting against tau neurotoxicity [59–62]. To determine whether XBP-1s modifies nuclear or cytoplasmic TDP-43 phenotypes, we crossed an established transgenic *xbp-1s* overexpression strain (*xbp-1s o/ex)* with TDP-43-Tg and TDP-43 ΔNLS to generate double transgenic strains TDP-43-Tg; *xbp-1s o/ex* and TDP-43 ΔNLS; *xbp-1s o/ex*. Using swimming as readout of neuronal dysfunction, we found that increased XBP-1s worsened motility in two independent TDP-43-Tg strains (Fig. 6A and Supplementary Fig. 6A) but improved two independent TDP-43 ΔNLS strains (TDP-43 ΔNLS (med) Fig. 6B, and TDP-43 ΔNLS (high) Supplementary Fig. 3B, C). To confirm *xbp-1s o/ex* was behaving consistently with prior published work [61, 63], we crossed it with a *C. elegans* model expressing wild-type human tau pan-neuronally (tau-Tg). In tau-Tg; *xbp-1s o/ex* animals, we saw a robust improvement in swimming (Fig. 6C), consistent with prior publications. Taken together, our data suggest that the location of toxic pTDP-43, whether it is in the nucleus or the cytoplasm, influences cellular responses and mechanisms of clearance (Fig. 6D).

## Discussion

Although TDP-43 can form cytoplasmic or nuclear pathologic protein inclusions in FTLD-TDP [1], little is known about whether disease processes differ based on localization of pathology. Expression of human TDP-43 in *C. elegans* neurons provides a tractable, gain-of-function system to dissect disease mechanisms [23, 24, 64]. However, previously generated strains accumulate TDP-43 predominantly in the nucleus. To address this question, we have developed new transgenic *C. elegans* models that accumulate cytoplasmic TDP-43, enabling comparative evaluations of nuclear versus cytoplasmic localized TDP-43.

In this study, we mutated the TDP-43 NLS to generate three *C. elegans* strains that accumulate predominantly cytoplasmic localized TDP-43. We compared these cytoplasmic TDP-43 strains to previously existing, well-characterized nuclear TDP-43 strains to identify similarities and differences in neurotoxic phenotypes, cellular responses, and protein homeostasis mechanisms. These data provide important insights into FTLD-TDP biology and represent a new system to better understand TDP-43 in disease.

From our characterization of TDP-43 ΔNLS strains, we found dose-dependent toxicity of cytoplasmic TDP-43 (Fig. 2). Interestingly, cytoplasmic TDP-43 appears to cause less severe motility deficits at equivalent protein levels than nuclear TDP-43. When there were similar amounts of cytoplasmic and nuclear TDP-43, the strains with nuclear TDP-43 had more severe motility defects, indicating greater neuronal dysfunction. However, burden of phosphorylated TDP-43 correlated with motility deficits similarly whether nuclear or cytoplasmic (Fig. 2). Likewise, loss-of-function mutation in *tax-6*, the *C. elegans* homolog of the TDP-43 phosphatase calcineurin, worsened both TDP-43-Tg and TDP-43 ΔNLS (med) strains and led to increased accumulation of phosphorylated TDP-43 (Fig. 5). In mouse models of TDP-43 proteinopathy, robust calcineurin depletion occurs during disease course [65, 66]. In patients with ALS, calcineurin activity is decreased [67, 68], supporting loss of this neuron resilience factor as a contributor to worsened disease outcomes. Our *C. elegans* data suggest that TDP-43 phosphorylation is toxic regardless of subcellular localization, and that calcineurin/ *tax-6* mechanisms controlling toxicity of phosphorylated TDP-43 are shared between the nucleus and the cytoplasm.

At high expression levels of cytoplasmic TDP-43, we observed severe neurodegeneration with prominent GABAergic neuronal loss, axonal defasciculation, gaps in the continuity of the nerve cord, and GFP-positive puncta along the nerve cord (Fig. 3). We found mild neurodegeneration in the medium expressing TDP-43 ΔNLS strain, with only about half of the worms developing detectable neuronal loss (Fig. 3A). Some of this neurodegeneration could be attributed to disrupted methylation, peroxide metabolism, or BMP signaling, which were found to be dysregulated in TDP-43 ΔNLS (med) animals by RNAseq.

When comparing RNA transcriptome data from nuclear versus cytoplasmic TDP-43 strains, we found few differentially expressed genes and no alterations to RNA splicing, supporting gain-of-function toxicity in this model independent of alterations to RNA metabolism. Transcription factor network analysis did suggest an opposite regulation of the transcription factor ZIP-2 between these strains, supporting distinct cellular responses to cytoplasmic versus nuclear TDP-43. In nuclear-localized TDP-43-Tg, ZIP-2 targets were decreased, but in cytoplasmic- localized TDP-43 ΔNLS, ZIP-2 targets were increased (Fig. 4). ZIP-2 is an immune response effector in *C. elegans* [69] that has also been shown to be activated due to age-related mitochondrial dysfunction in *C. elegans* and stalled translation elongation [51, 70]. TDP-43 has been previously implicated in mitochondrial health and function [71–73]. However, a partial deletion of *zip-2* failed to impact motility phenotypes of TDP-43-Tg or TDP-43 ΔNLS. In this case, *zip-2* target regulation in TDP-43 expressing strains may be indicative of cellular responses downstream of phenotypic drivers, but which do not in themselves promote neuronal dysfunction.

ER stress can be triggered by the accumulation of misfolded or unfolded proteins in the cytoplasm and in the ER lumen, resulting in activation of the unfolded protein response (UPR^ER^). During activation of the UPR^ER^, XBP1 mRNA becomes alternatively spliced into the activated form XBP1s, which then increases transcription of genes related to degrading misfolded proteins [74]. Reduced XBP-1 exacerbates neurotoxicity in tau models of *D. melanogaster* [59] and *C. elegans* [60], while overexpressing XBP-1s is neuroprotective in tau models of *C. elegans* [61, 63]. Interestingly, we found divergent effects of *xbp-1s* overexpression on nuclear versus cytoplasmic expressing TDP-43 strains. *xbp-1s* suppressed neurotoxic motility phenotypes of cytoplasmic TDP-43 ΔNLS strains, but worsened motility of nuclear TDP-43-Tg strains (Fig. 6, Supplementary Fig. 6). The neuroprotection against cytoplasmic TDP-43 aligns with its ability to protect against tau, another cytoplasmic accumulating aggregating protein. However, it is not clear why increased *xbp-1s* results in worsened outcomes for strains with nuclear-localized TDP-43. It is possible that an *xbp-1s*-mediated increase in cytoplasmic protein clearance decreases cellular ability to respond and clear nuclear proteins. This contrast between *xbp-1s* overexpression driving either a protective or worsened reaction based on the location of TDP-43 in the cell highlights potential differences in disease mechanisms or cellular responses to pathological variation in human disease. Nuclear versus cytoplasmic TDP-43 inclusions may be physical manifestations of differences in disease etiology, progression, or cellular responses among individuals critical for effective treatments. The *C. elegans* models described here provide a comparative study of outcomes from TDP-43 pathologic localization, enabling a greater understanding of TDP-43 in normal and disease states essential for the development of effective therapies.

## Supplementary information

Supplemental Figure Legends, Supplemental Figures 1-6, Supplemental Table 1

Supplemental Tables 2-8

Complete Western blots

## Data Availability

There are no restrictions on the availability of datasets contained within this paper. Original data are contained within the main manuscript and supporting files and are available upon request. RNAseq data is available at GEO accession GSE326885.
