# Supplementary material for: Shared and distinct gain-of-function consequences from pathologic cytoplasmic versus nuclear TDP-43
Source: Cell Death Dis. 2026 Aug 11;17(1):944. doi: 10.1038/s41419-026-09143-9 (PMC13740324; doi:10.1038/s41419-026-09143-9)
Supplement: Supplementary file 1 [file 41419_2026_9143_MOESM1_ESM.pdf]

Sup. Fig. 1. pTDP-43 is localized in the cytoplasm and not the nucleus in TDP-43 ∆NLS worms.
(A) Immunostaining for nuclei (DAPI) and phosphorylated TDP-43 (pTDP-43) in day 1 adult TDP-43 ∆NLS (med) *C. elegans*. Size bar = 10μm.

Sup. Fig. 2. TDP-43 is visible in TDP-43 ∆NLS (low) but not non-Tg control in long exposure.
(A) Synchronized day 1 adult *C. elegans* were tested by immunoblot for total TDP-43, and left overnight to maximize signal on film. TDP-43 is visible in all the lanes other than the non-Tg lane.

Sup. Fig. 3. *tdp-1 KO* does not alter motility in nuclear or cytoplasmic TDP-43 strains.
Motility of *tdp-1 KO* day 1 adults was measured by radial locomotion (linear distance crawling in one hour on an agar plate). Neither TDP-43-Tg 3 nor TDP-43 ∆NLS (med) strains exhibited altered motility in the presence of *tdp-1(bk3053)*. One-way ANOVA with Tukey's multiple comparison test. ****p<0.0001. ns, not significant. Data are mean ± s.e.m.

Sup. Fig. 4. (A) Principal component analysis of gene expression in TDP-43 ΔNLS (med) Tg, TDP-43-Tg, and non-Tg controls (n = 12). **(B)** A volcano plot of differential RNA splicing in TDP-43 ΔNLS (med) Tg animals compared to non-Tg controls (n =8). **(C)** A volcano plot of differential RNA splicing in TDP-43-Tg animals compared to non-Tg controls (n =8). **(D)** A volcano plot of differential RNA splicing in tdp-1(csb38) null animals compared to non-Tg controls re-analyzed from (53), (n =6).

Sup. Fig. 5. *zip-2(ok3730)* does not alter motility in nuclear or cytoplasmic TDP-43 strains.
Motility of *zip-2(ok3730)* day 1 adults was measured by **(A)** radial locomotion (linear distance crawling in one hour on an agar plate) and **(B)** swimming in liquid. Neither TDP-43-Tg nor TDP-43 ∆NLS (high) strains exhibited altered motility in the presence of *zip-2(ok3730)*. One-way ANOVA with Tukey's multiple comparison test. ns, not significant. Data are mean ± s.e.m.

Sup. Fig. 6. *xbp-1s* overexpression protects against cytoplasmic TDP-43 but worsens outcomes for nuclear TDP-43 strains. (A-C) *xbp-1s o/ex* driven motility changes of day 1 adults as measured by swimming (rates of thrashing in liquid for a minute) and radial locomotion (linear distance crawling in one hour on an agar plate). *xbp-1s o/ex* **(A)** worsened swimming in

TDP-43-Tg (low) but improves swimming in **(B)** TDP-43 ΔNLS (high). *xbp-1s o/ex* **(C)** improves radial locomotion in TDP-43 ΔNLS (med). One-way ANOVA with Tukey's multiple comparison test. **p<0.01, ***p<0.001, ****p<0.0001. ns, not significant. Data are mean ± s.e.m.

Table S1. Complete list of all strains and descriptions used in this study

Table S2. Differential gene expression in TDP-43 ΔNLS (med) animals.

Table S3. Differential estimated transcription factor activity in TDP-43 ΔNLS (med) animals.

Table S4. Differential gene expression in TDP-43-Tg animals.

Table S5. Differential estimated transcription factor activity in TDP-43-Tg animals.

Table S6. Differential RNA splicing in TDP-43 ΔNLS (med) animals.

Table S7. Differential RNA splicing in TDP-43-Tg animals.

Table S8. Differential RNA splicing in *tdp-1(csb38)* null animals.

A

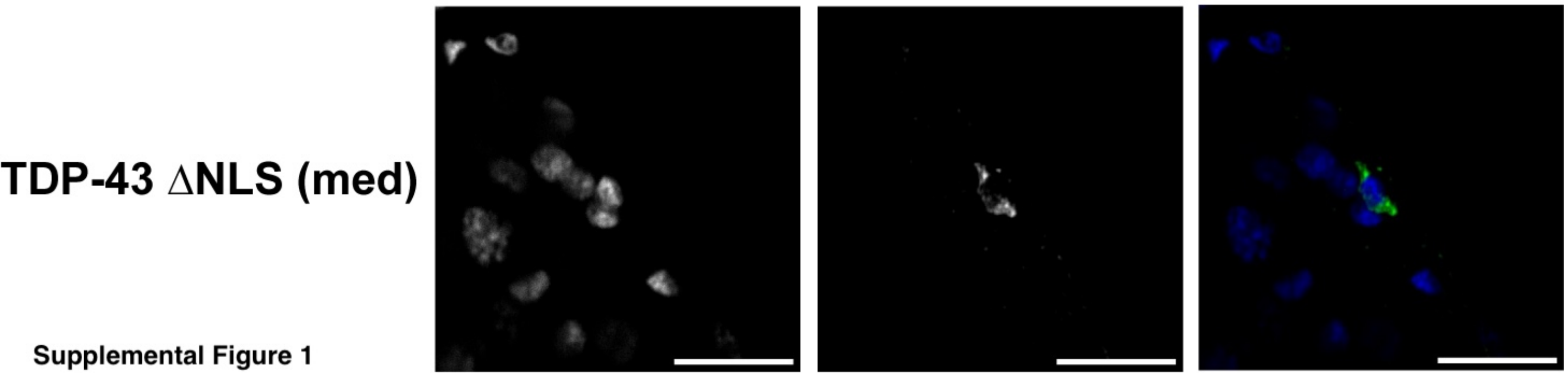

Supplemental Figure 1

A

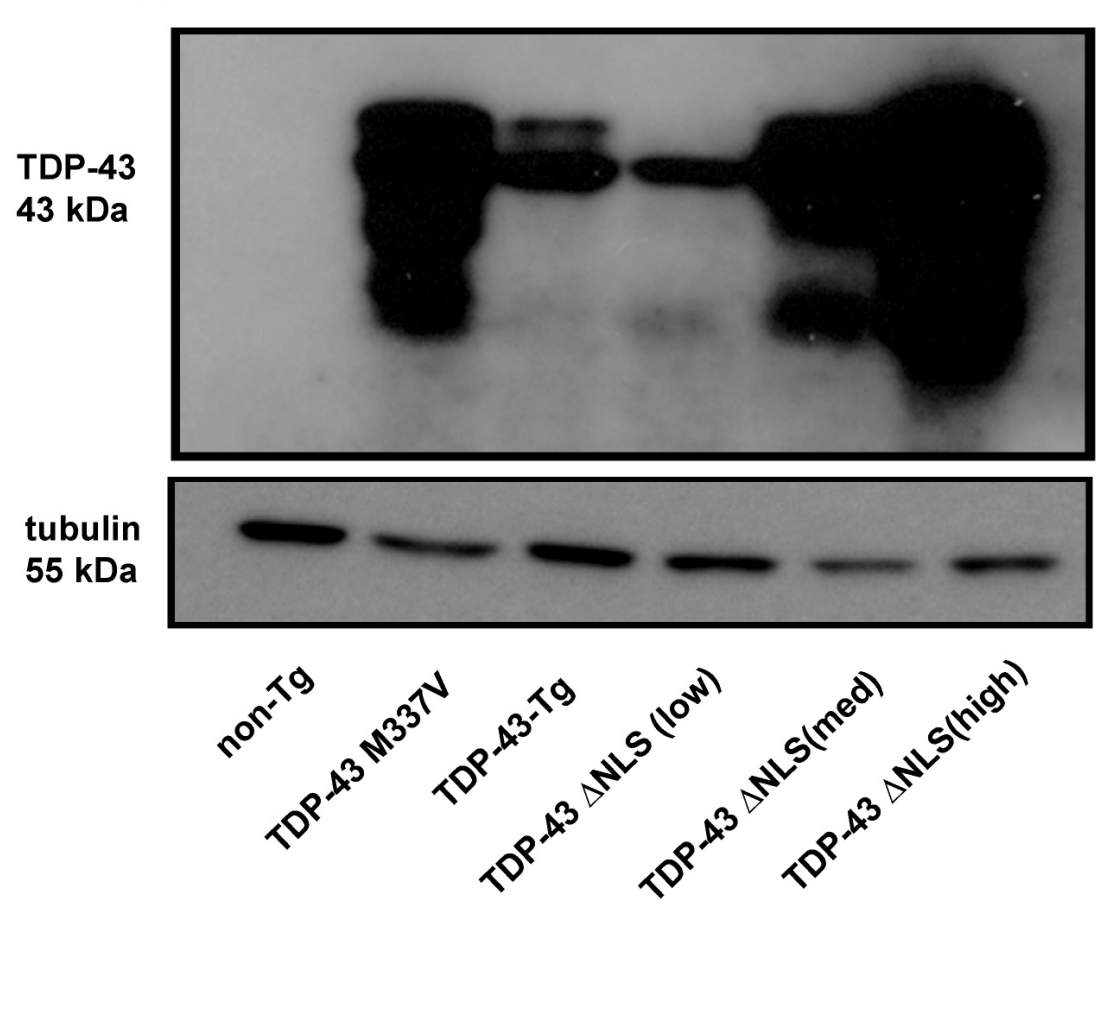

Supplemental Figure 2

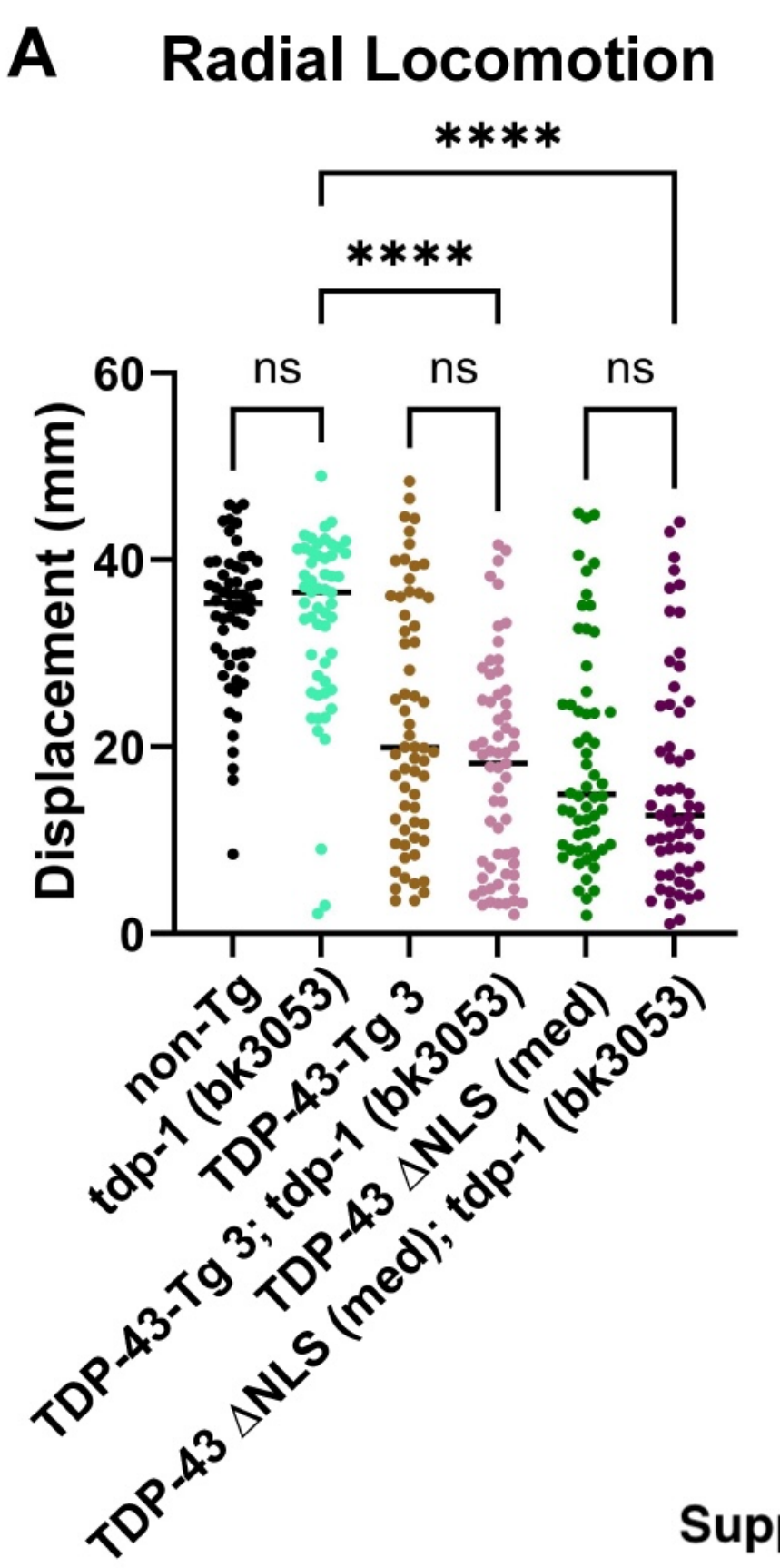

Supplemental Figure 3

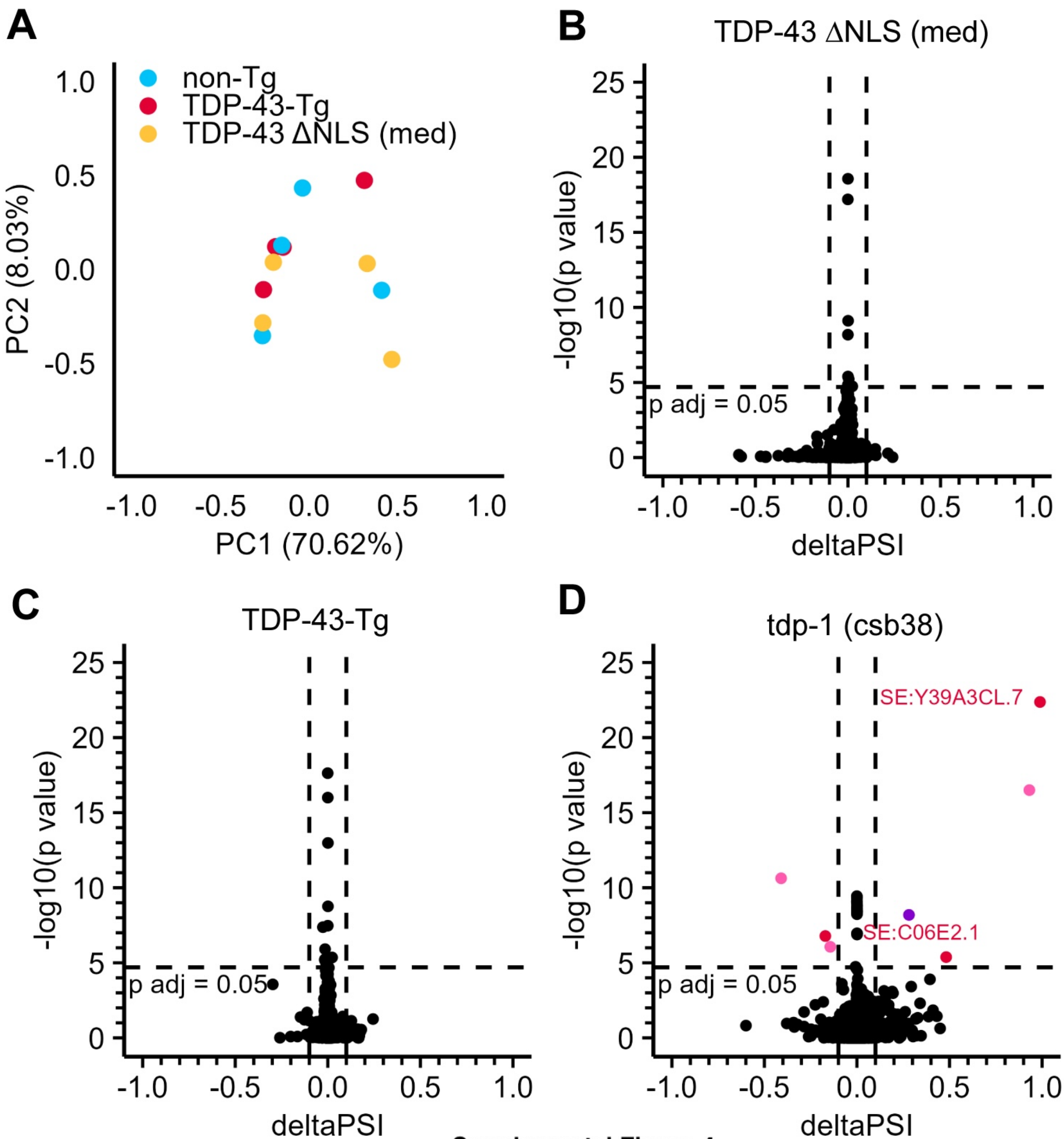

Supplemental Figure 4

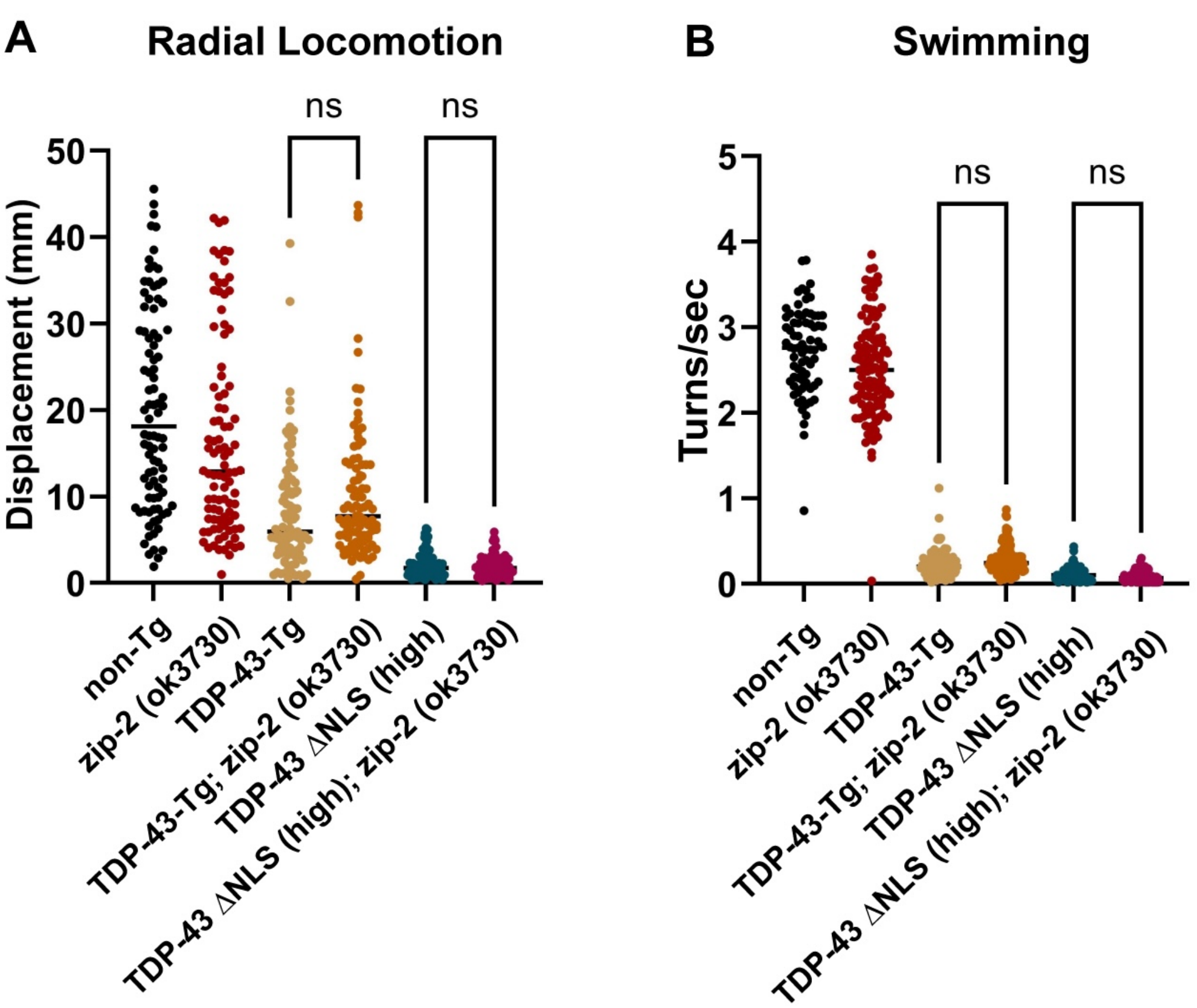

Supplemental Figure 5

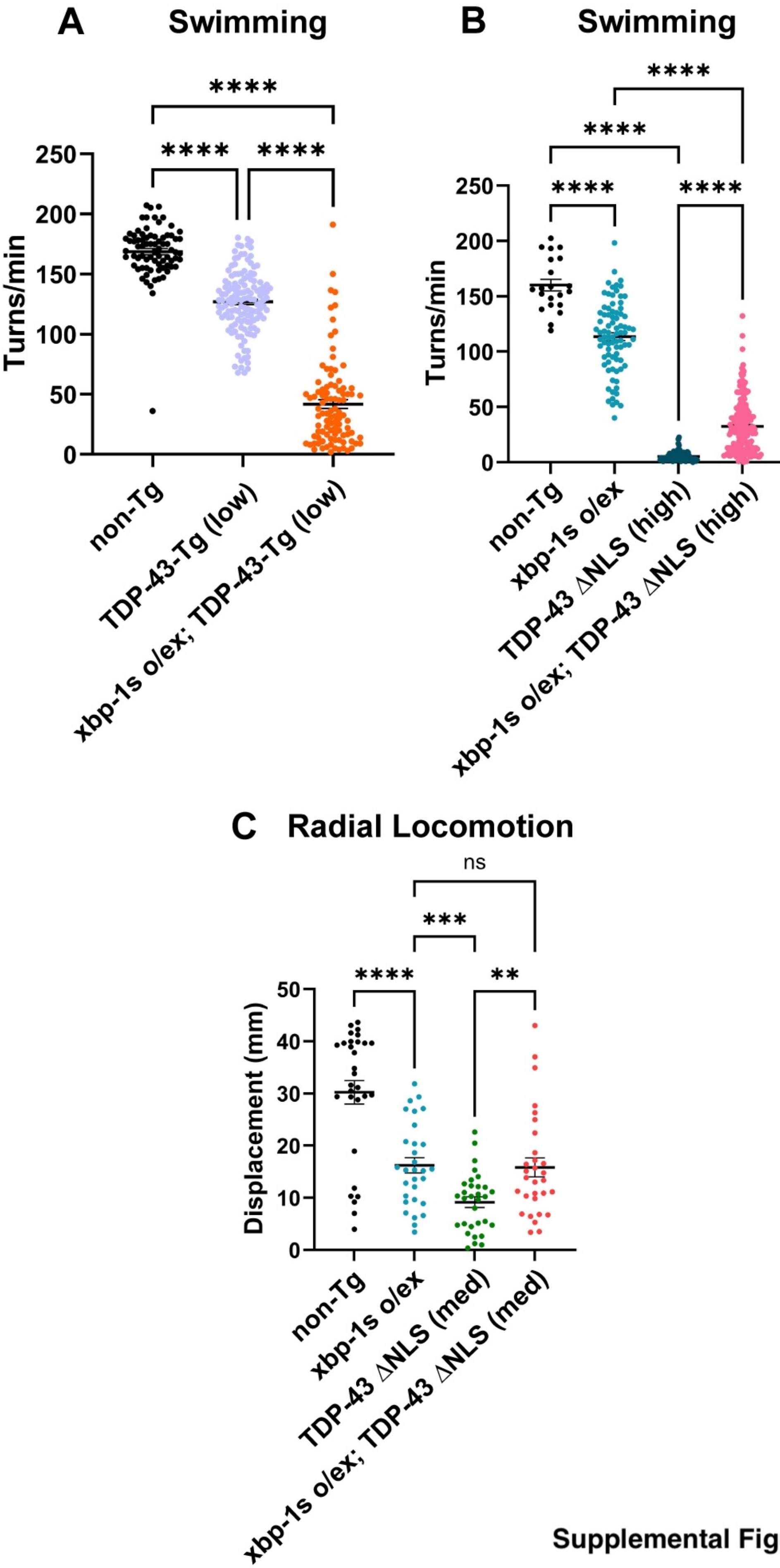

Supplemental Figure 6

Supplemental Table 1

Strain abbreviations	Strain name	Strain genotype
non-Tg	N2	Wildtype
TDP-43 ΔNLS (low)	NLS152	*liaIs152[Psnb-1::TDP-43 (K82A/R83A/K84A) + Pmyo-3::dsRED]*
TDP-43 ΔNLS (med)	NLS151	*liaIs151[Psnb-1::TDP-43 (K82A/R83A/K84A) + Pmyo-3::dsRED]*
TDP-43 ΔNLS (high)	NLS142	*liaIs142[Psnb-1::TDP-43 (K82A/R83A/K84A) + Pmyo-3::dsRED]*
TDP-43-Tg	CK410	*bkIs410[Psnb-1::TDP-43; Pmyo-2::dsRED + Pmyo-2::dsRED]*
TDP-43 M337V	CK423	*bkIs423[Psnb-1::TDP-43 (M337V) + Pmyo-2::dsRED]*
CZ1200/control	CZ1200	*juIs76[unc-25p::GFP + lin-15(+) II.]*
CZ1200; TDP-43 ΔNLS (med)	CZ1200; NLS151	*juIs76[unc-25p::GFP + lin-15(+)] II] ;liaIs151[Psnb-1::TDP-43 (K82A/R83A/K84A) + Pmyo-3::dsRED]*
CZ1200; TDP-43 ΔNLS (high)	CZ1200; NLS142	*juIs76[unc-25p::GFP + lin-15(+)] II]; liaIs142[Psnb-1::TDP-43 (K82A/R83A/K84A) + Pmyo-3::dsRED]*
TDP-43-Tg 2	CK403	*bkIs403[Psnb-1::TDP-43 + Pmyo-3::GFP]*
TDP-43-Tg (low)	CK1943	*bkIs1943[Psnb-1::hTDP-43 (WT)::K4aptazyme:unc-54 3'UTR + Pmyo-3::mCherry]*
tax-6 KO	RB1667	*tax-6(ok2065)*
TDP-43 ΔNLS (med); tax-6 KO	NLS220	*tax-6(ok2065); liaIs151[Psnb-1::TDP-43 (K82A/R83A/K84A) + Pmyo-3::dsRED]*
TDP-43-Tg; tax-6 KO	NLS221	*tax-6(ok2065); bkIs410[Psnb-1::TDP-43 + Pmyo-3::dsRED]*
tax-6 GOF	KJ306	*tax-6(jh107)*
TDP-43 ΔNLS (med); tax-6 GOF	NLS268	*tax-6(jh107); liaIs151[Psnb-1::TDP-43 (K82A/R83A/K84A) + Pmyo-3::dsRED]*
TDP-43-Tg; tax-6 GOF	NLS269	*tax-6(jh107); bkIs410[Psnb-1::TDP-43 + Pmyo-3::dsRED]*
xbp-1s o/ex	AGD927	*uthIs270[Prab-3::xbp-1s+ Pmyo-2::tdTomato]*
TDP-43 ΔNLS (med); xbp-1s o/ex	NLS234	*uthIs270[Prab-3::xbp-1s+ Pmyo-2::tdTomato]; liaIs151[Psnb-1::TDP-43 (K82A/R83A/K84A) + Pmyo-3::dsRED]*
TDP-43 ΔNLS (high); xbp-1s	CK2961	*uthIs270[Prab-3::xbp-1s+ Pmyo-2::tdTomato]; liaIs142[Psnb-1::TDP-43(K82A/R83A/K84A) + Pmyo-3::dsRED]*
TDP-43-Tg-2 ; xbp-1s o/ex	CK2994	*uthIs270[Prab-3::xbp-1s+ Pmyo-2::tdTomato]; bkIs403[Psnb-1::TDP-43 + Pmyo-3::GFP]*
TDP-43-Tg (low); xbp-1s o/ex	CK1943;AGD927	*uthIs270[Prab-3::xbp-1s+ Pmyo-2::tdTomato]; bkIs1943[Psnb-1::hTDP-43 (WT)::K4aptazyme:unc-54 3'UTR + Pmyo-3::mCherry]*
tau-Tg; xbp-1s o/ex	CK933	*uthIs270[Prab-3::xbp-1s+ Pmyo-2::tdTomato]; bkIs144[Paex-3::Tau WT (4R1N) + Pmyo-2::GFP]*
tau-Tg	CK144	*bkIs144[Paex-3::Tau WT (4R1N) + Pmyo-2::GFP]*
zip-2(ok3730)	VC3056	*zip-2(ok3730)*
TDP-43 ΔNLS (high); zip-2(ok3730)	NLS271	*zip-2(ok3730); liaIs142[Psnb-1::TDP-43 (K82A/R83A/K84A) + Pmyo-3::dsRED]*
TDP-43-Tg; zip-2(ok3730)	NLS272	*zip-2(ok3730); bkIs410[Psnb-1::TDP-43 + Pmyo-3::dsRED]*
TDP-43-Tg 3	CK402	*bkIs402[Psnb-1::TDP-43 + Pmyo-2::GFP]*
tdp-1 KO	CK3053	*tdp-1(bk3053)*
TDP-43 ΔNLS (med); tdp-1 KO	NLS273	*tdp-1(bk3053); liaIs151[Psnb-1::TDP-43 (K82A/R83A/K84A) + Pmyo-3::dsRED]*
TDP-43-Tg 3; tdp-1 KO	NLS274	*tdp-1(bk3053); bkIs402[Psnb-1::TDP-43 + Pmyo-2::GFP]*
