# Supplementary material for: Shared and distinct gain-of-function consequences from pathologic cytoplasmic versus nuclear TDP-43
Source: Cell Death Dis. 2026 Aug 11;17(1):944. doi: 10.1038/s41419-026-09143-9 (PMC13740324; doi:10.1038/s41419-026-09143-9)

Total TDP-43 5 min exposure

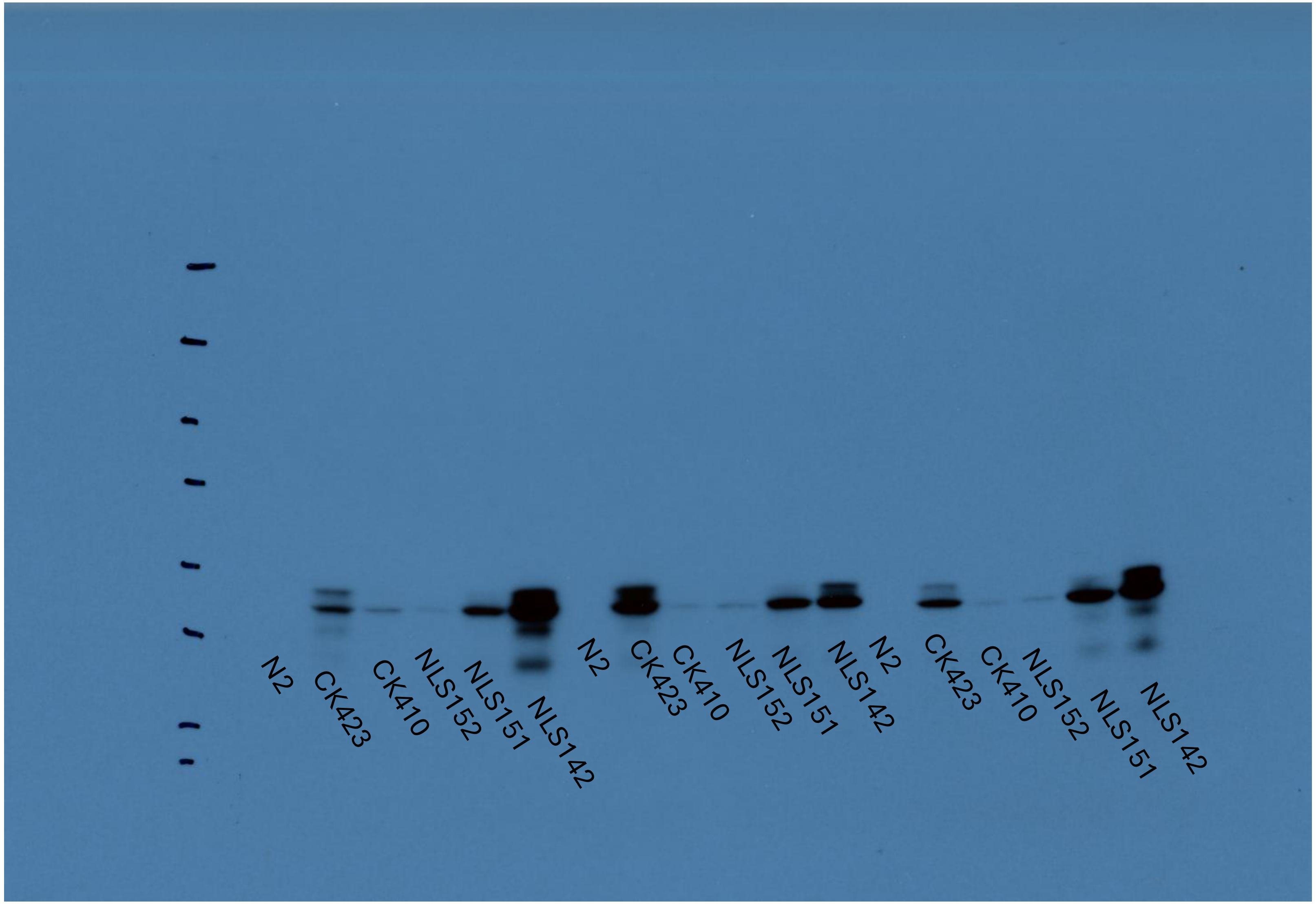

Total TDP-43 overnight exposure

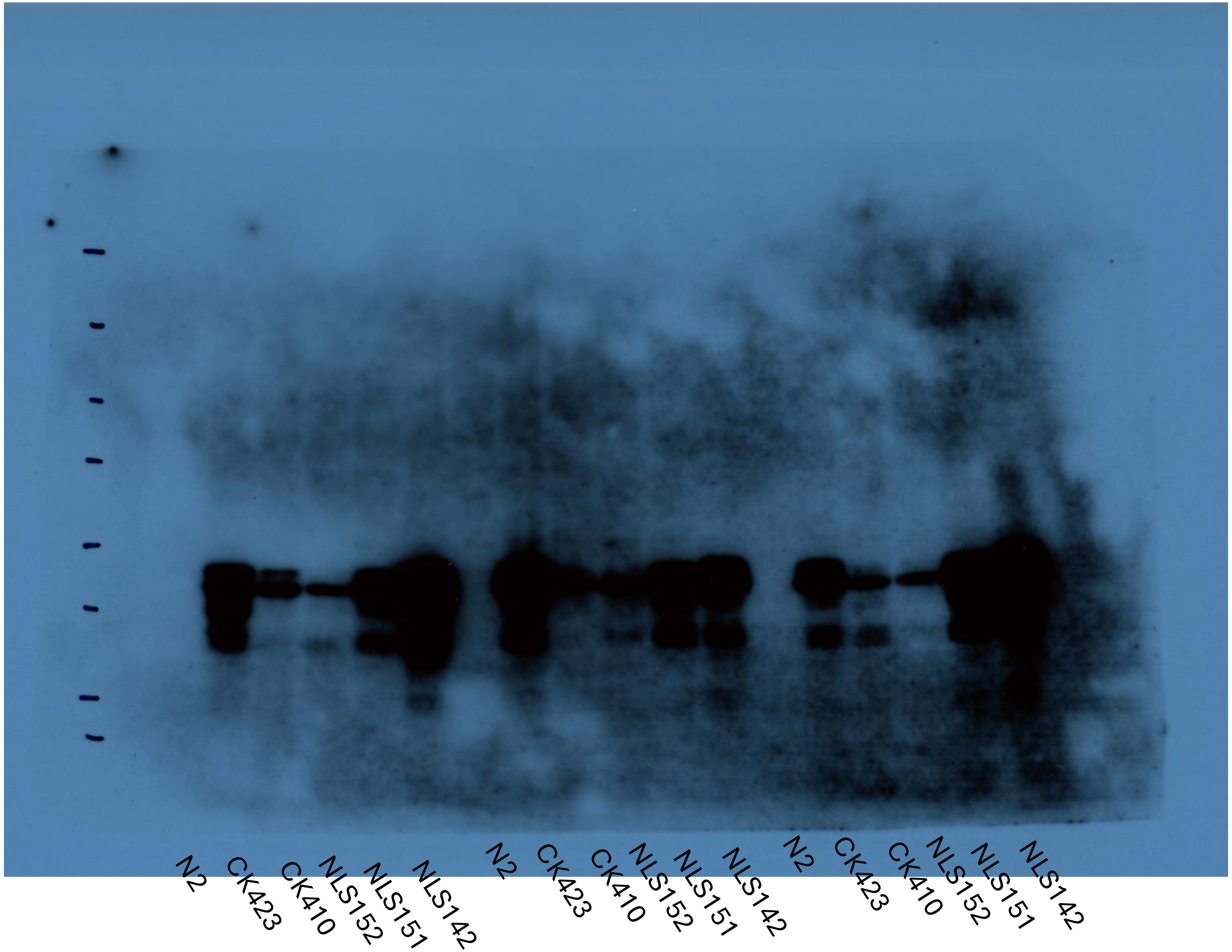

Tubulin 30 second exposure

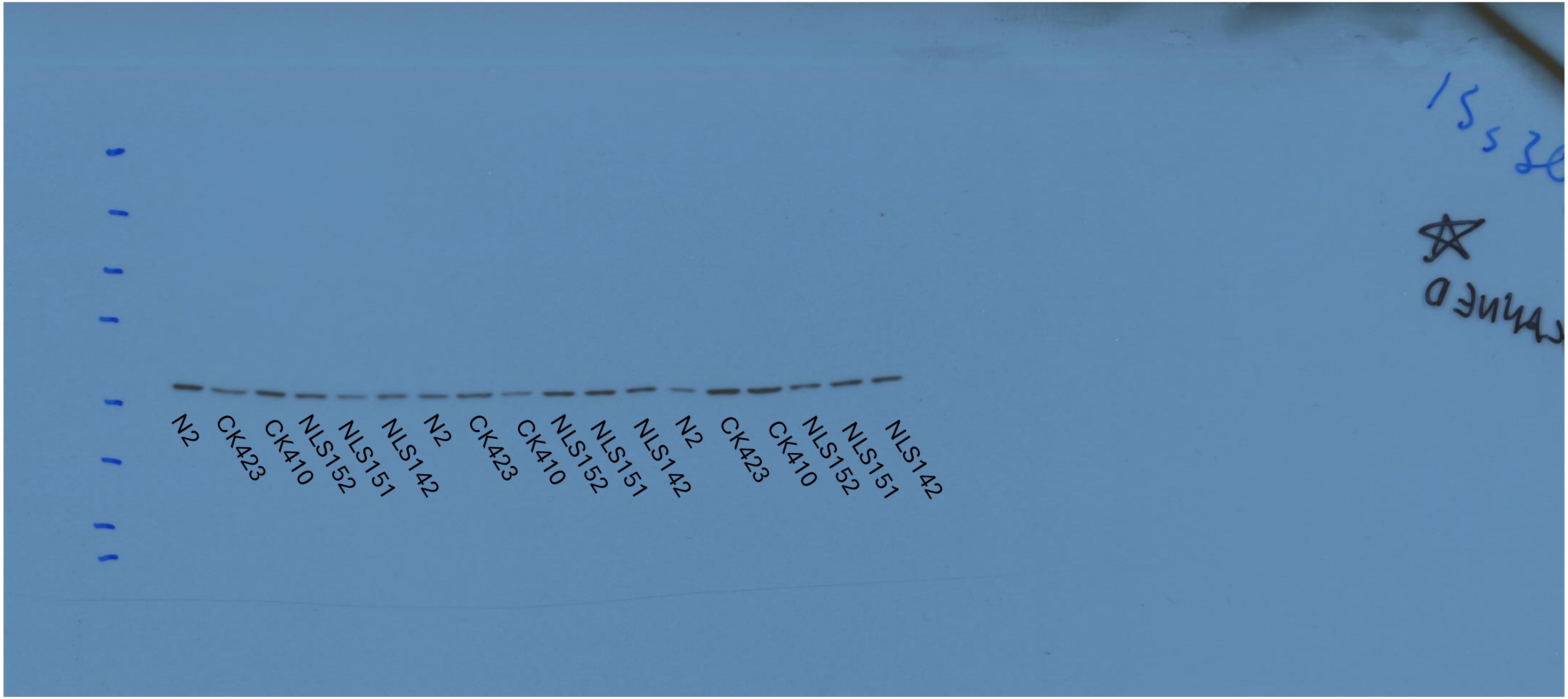

Phospho TDP-43 10 min exposure

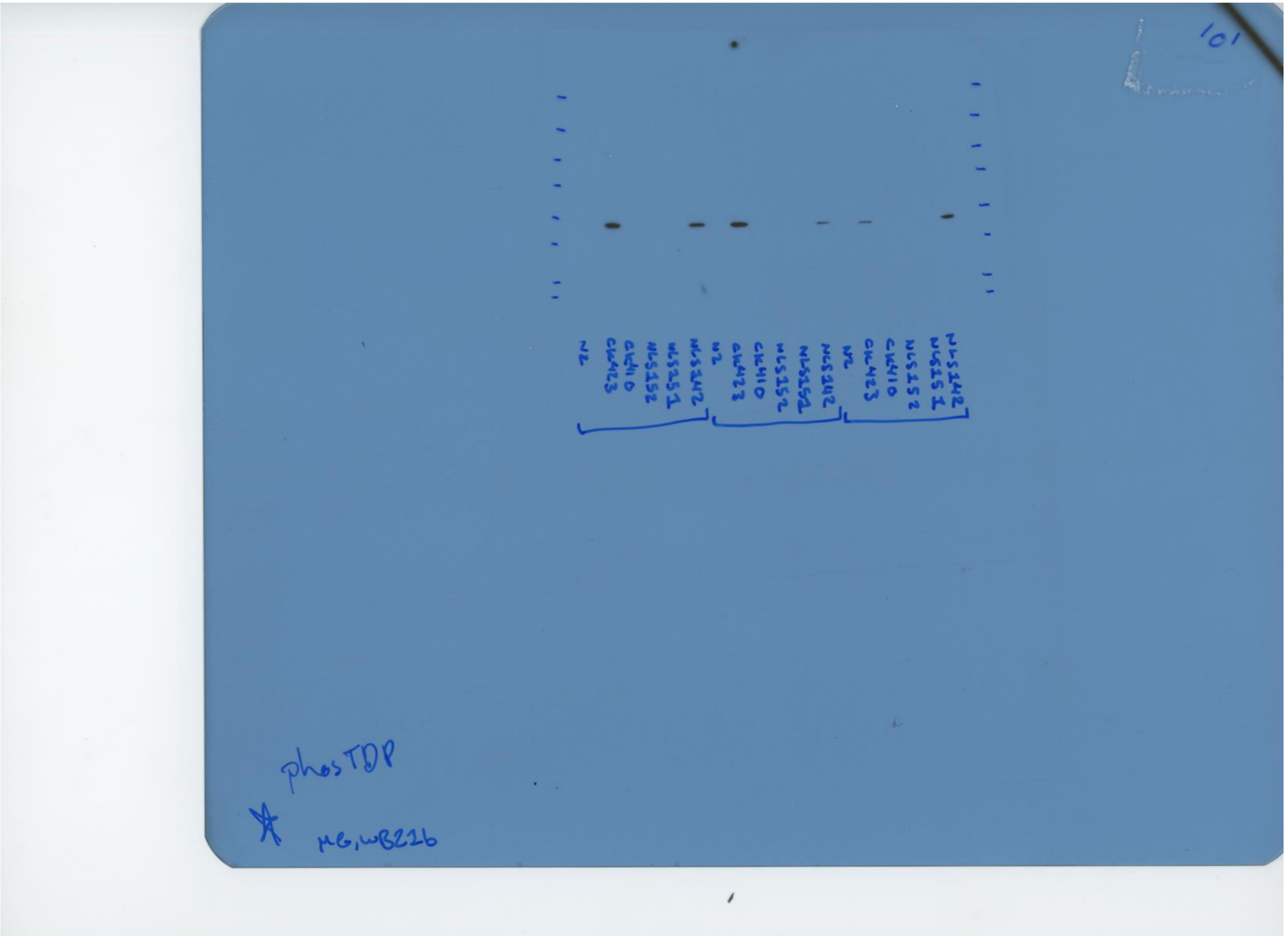

Phospho TDP-43 overnight exposure

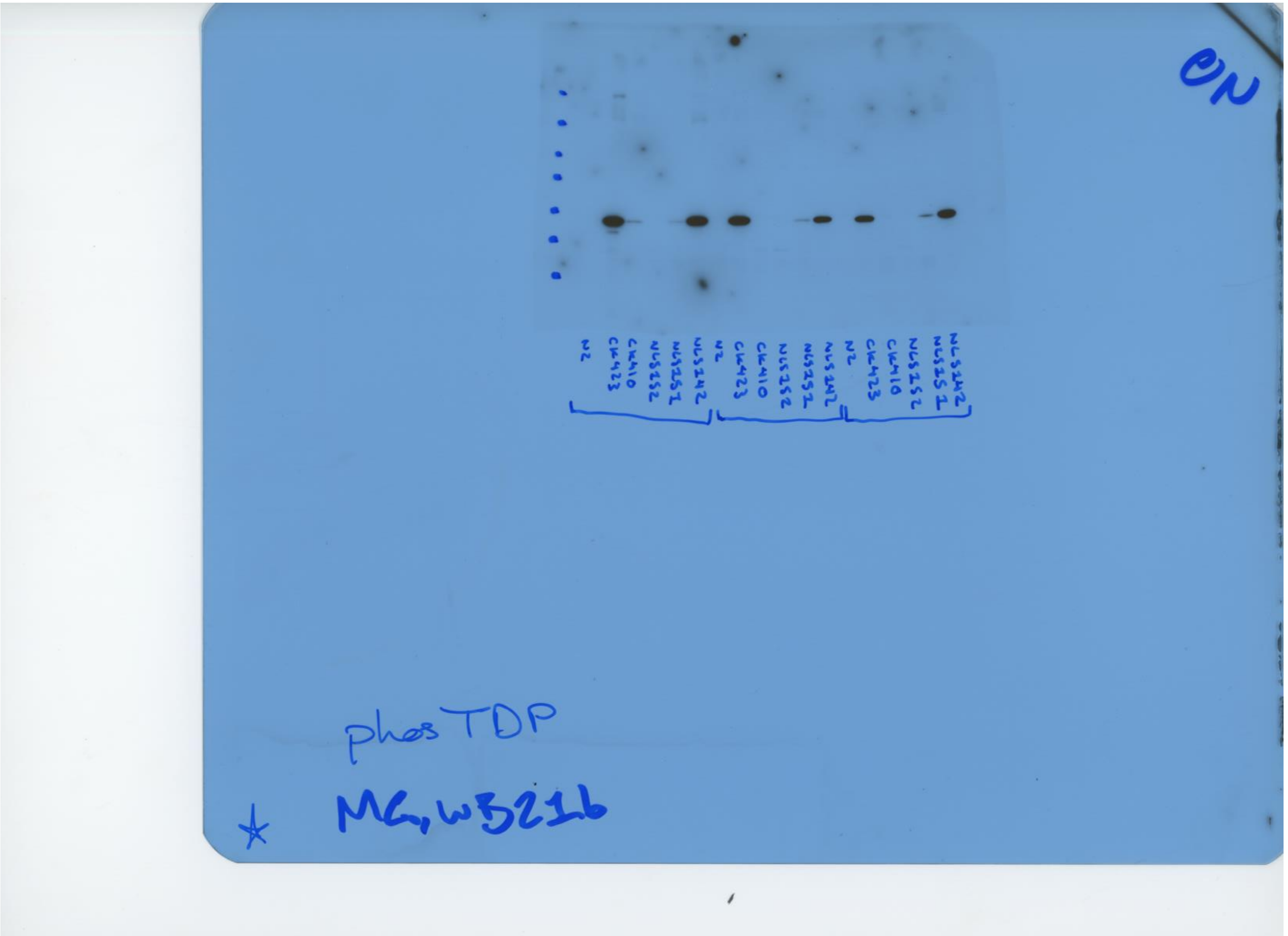

Tax-6 KO tubulin 15 second exposure

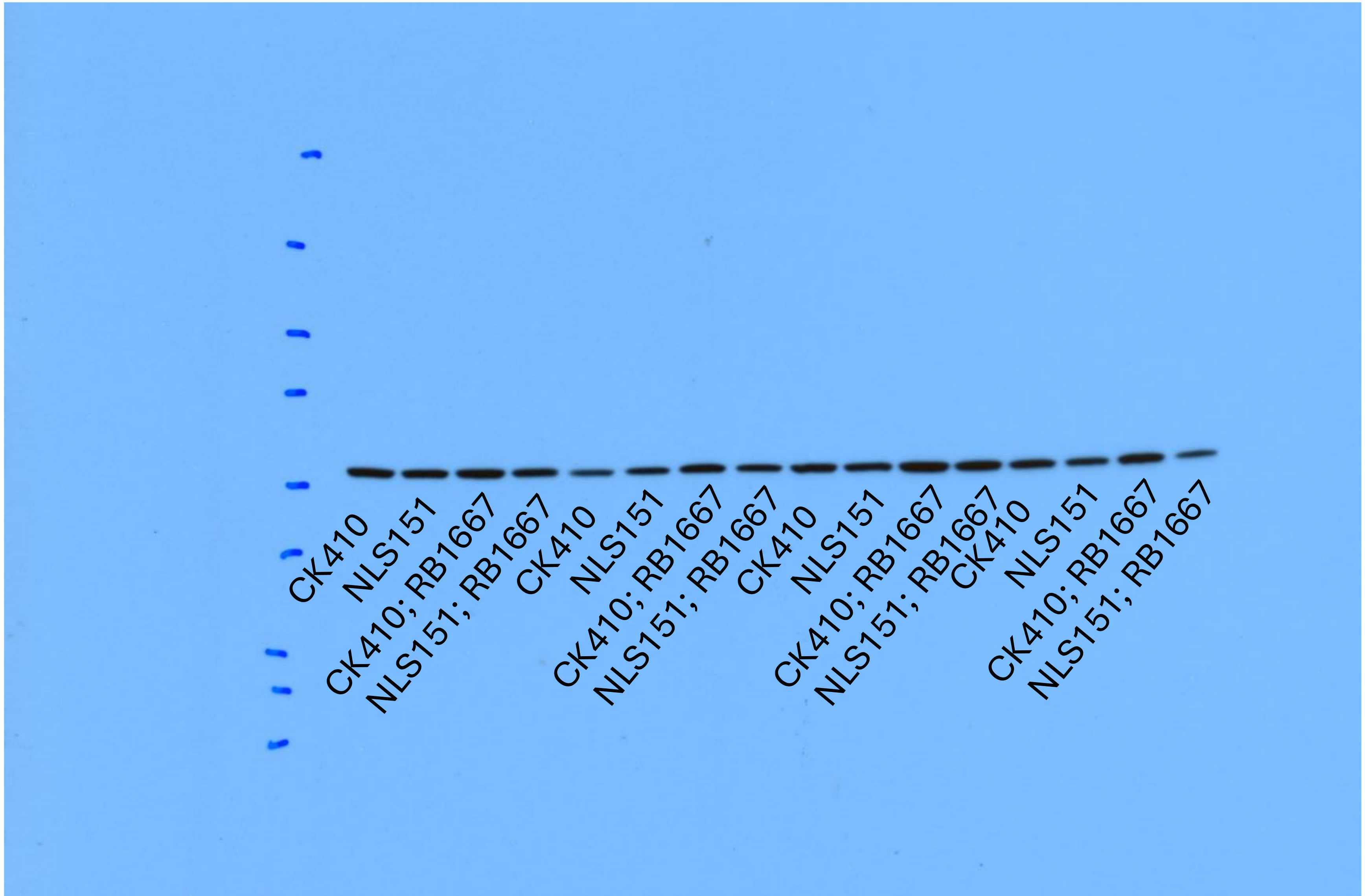

Tax-6 KO tubulin and TDP-43 5min exposure

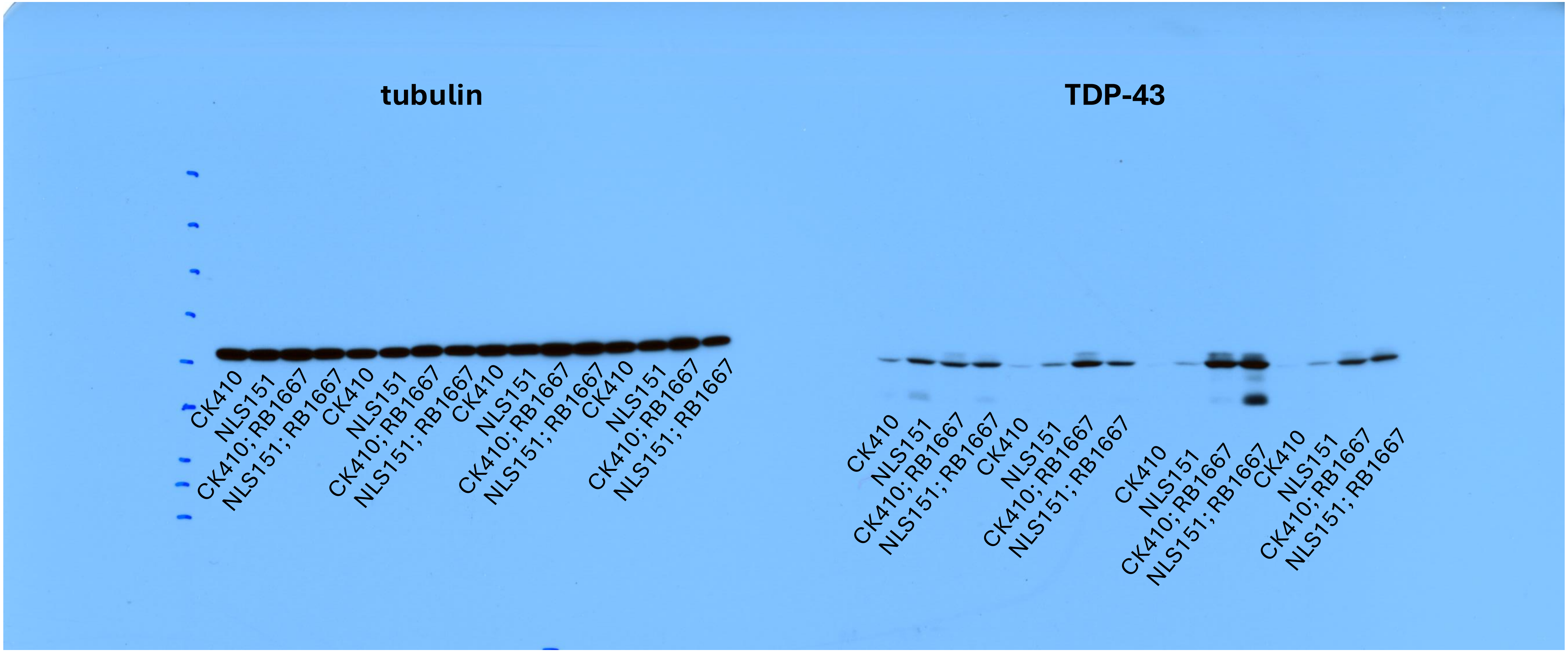

Tax-6 LOF pTDP-43 5min exposure

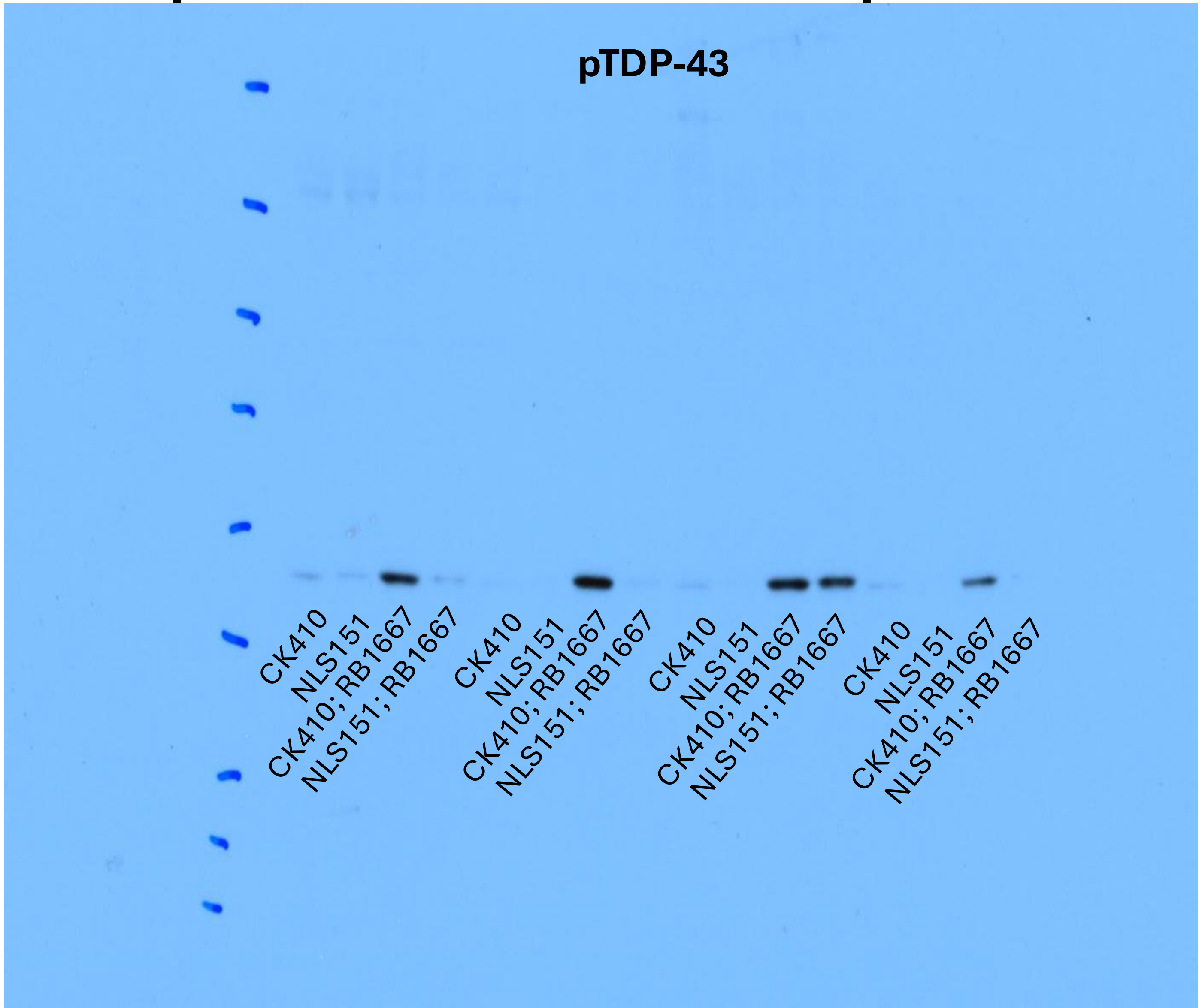

Tax-6 KO TDP-43 and pTDP-43 10min exposure

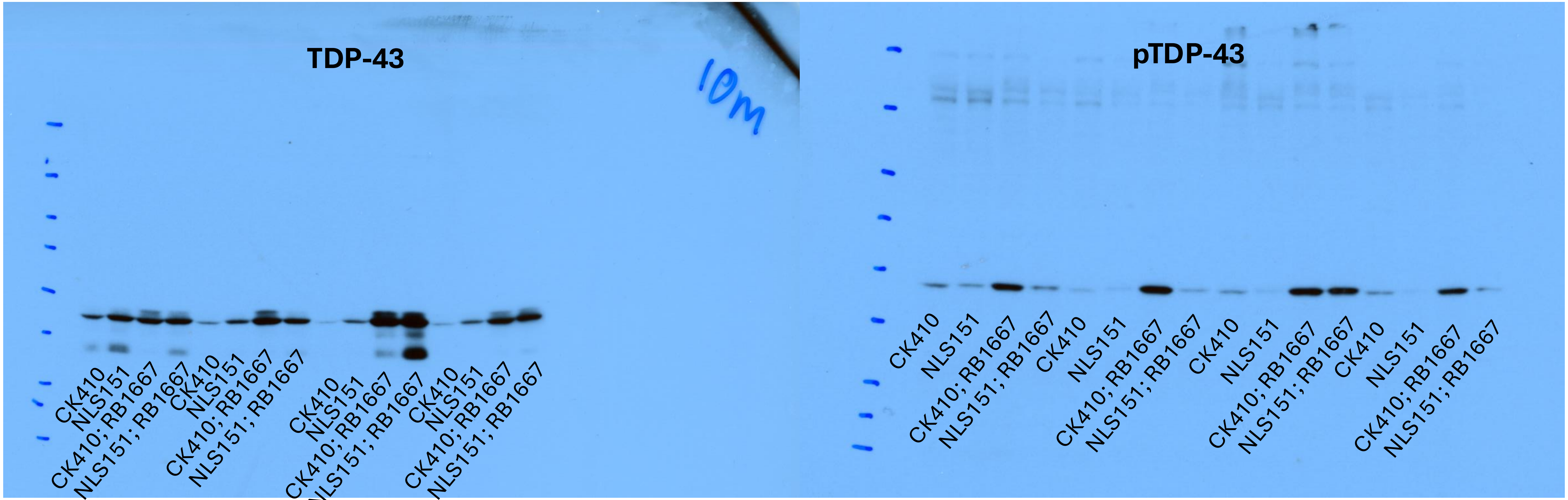

Tax-6 KO TDP-43 and pTDP-43 30min exposure

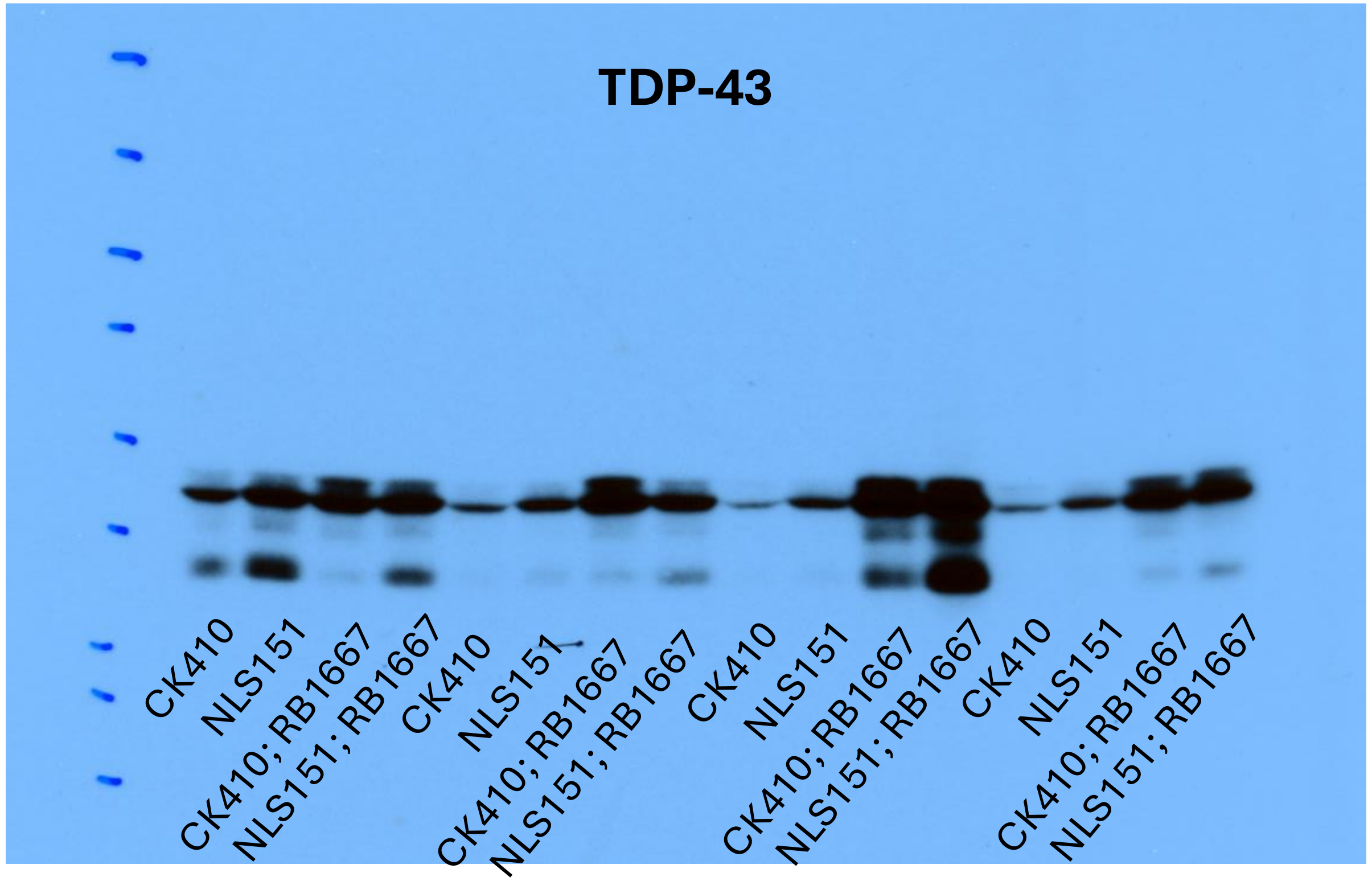

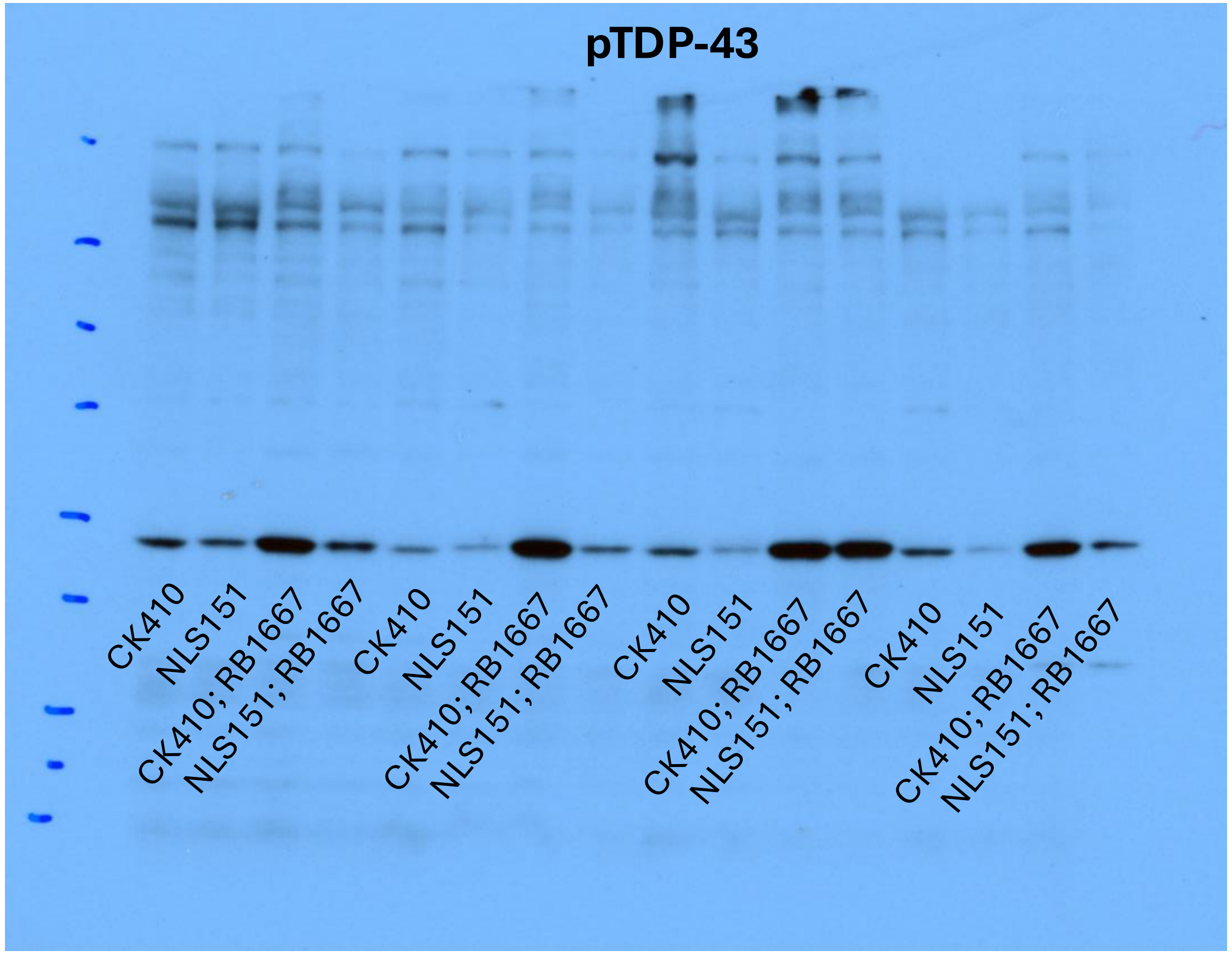

Supplement: Supplementary file 2 [file 41419_2026_9143_MOESM3_ESM.pdf]
